# A systematic review and meta-analysis of preanalytical factors and methodological differences influencing the measurement of circulating vascular endothelial growth factor

**DOI:** 10.1371/journal.pone.0270232

**Published:** 2022-07-06

**Authors:** Ulrika Sjöbom, Anders K. Nilsson, Hanna Gyllensten, Ann Hellström, Chatarina Löfqvist

**Affiliations:** 1 Learning and Leadership for Health Care Professionals At the Institute of Health and Care Science at Sahlgrenska Academy at University of Gothenburg, Gothenburg, Sweden; 2 Department of Clinical Neuroscience At the Institution of Neuroscience and Physiology at Sahlgrenska Academy at University of Gothenburg, Gothenburg, Sweden; University of Rochester, UNITED STATES

## Abstract

**Background:**

Intraocular treatment with antibodies targeting vascular endothelial growth factor (anti-VEGF) inhibits pathological vessel growth in adults and preterm infants. Recently, concerns regarding the impact of anti-VEGF treatment on systemic VEGF levels in preterm infants have been raised. Earlier studies suggest that preanalytical and methodological parameters impact analytical VEGF concentrations, but we have not found a comprehensive systematic review covering preanalytical procedures and methods for VEGF measurements.

**Objective:**

This review aimed to evaluate the most critical factors during sample collection, sample handling, and the analytical methods that influence VEGF levels and therefore should be considered when planning a prospective collection of samples to get reproducible, comparable results.

**Material and methods:**

PubMed and Scopus databases were searched 2021/Nov/11. In addition, identification of records via other methods included reference, citation, and Google Scholar searches. Rayyan QCRI was used to handle duplicates and the selection process. Publications reporting preanalytical handling and/or methodological comparisons using human blood samples were included. Exclusion criteria were biological, environmental, genetic, or physiological factors affecting VEGF. The data extraction sheets included bias assessment using the QUADAS-2 tool, evaluating patient selection, index-test, reference standard, and flow and timing. Concentrations of VEGF and results from statistical comparisons of analytical methods and/or preanalytical sample handling and/or different sample systems were extracted. The publications covering preanalytical procedures were further categorized based on the stage of the preanalytical procedure. Meta-analysis was used to visualize VEGF concentrations among healthy individuals. The quality of evidence was rated according to GRADE.

**Results:**

We identified 1596 publications, and, after the screening process, 43 were considered eligible for this systematic review. The risk of bias estimation was difficult for 2/4 domains due to non-reported information. Four critical steps in the preanalytical process that impacted VEGF quantification were identified: blood drawing and the handling before, during, and after centrifugation. Sub-categorization of those elements resulted in nine findings, rated from moderate to very low evidence grade. The choice of sample system was the most reported factor. VEGF levels (mean [95% CI]) in serum (n = 906, 20 publications), (252.5 [213.1–291.9] pg/mL), were approximated to ninefold higher than in plasma (n = 1122, 23 publications), (27.8 [23.6–32.1] pg/mL), based on summarized VEGF levels with meta-analysis. Notably, most reported plasma levels were below the calibration range of the used method.

**Conclusion:**

When measuring circulating VEGF levels, choice of sample system and sample handling are important factors to consider for ensuring high reproducibility and allowing study comparisons. Protocol: CRD42020192433

## 1. Introduction

The formation of new blood vessels, termed angiogenesis, is a fundamental process observed during embryonal development as well as in the course of several diseases, such as tumor development, metastasis, and retinal disorders. The essential role of vascular endothelial growth factor (VEGF) in angiogenesis [1] has been extensively studied because of its importance for tumor vessel growth [2]. Moreover, VEGF is essential for embryonic development [3] and the pathogenesis of preeclampsia [4]. Four different genes express VEGF in variants VEGF- A, B, C, and D [5]. VEGF-A is the most studied variant and is the one referred to in this paper unless otherwise specified.

Because of the pro-angiogenetic properties of VEGF, treatment strategies involving binding to VEGF and thereby inhibiting the effect of VEGF via its receptor have been developed. These so-called anti-VEGF drugs, like Avastin®, were first developed and used together with cytostatics to treat colorectal cancer [6–8], aiming to decrease the vessel growth in the tumor. In the last fifteen years, intraocular anti-VEGF drugs have been employed to arrest vessel growth when treating retinal and choroidal vascular diseases [9]. In recent years, this treatment approach has also been used to inhibit pathological vessel growth in the treatment of retinopathy of prematurity (ROP) in preterm infants [10].

ROP is a sight-threatening disease with a risk for retinal detachment and blindness if severe cases are not identified and treated [10]. The disease primarily affects prematurely born infants: the highest risk of ROP is observed among infants born before 30 postmenstrual weeks [11]. The disease develops in two distinct phases, and VEGF plays essential roles in both. In the first phase, angiogenesis is disrupted by the high oxygen exposure after birth [12], and VEGF in the retina decreases leading to vessel arrest [13]. In the second phase, VEGF increases and induce abnormal vessel growth [12]. Intra-ocular injections with anti-VEGF in the second phase of ROP seem to exert a similar therapeutic effect on the disease as the standard treatment with laser photocoagulation [14–16]. However, there is evidence for systemic leakage of intraocularly injected anti-VEGF [17, 18]. In utero, fetal growth is extensive during the third trimester, which corresponds to the first months of life for infants born extremely preterm. Within this period, organs such as the lungs [19] and the brain [20] are still undergoing the process of maturation, and anti-VEGF-mediated inhibition of blood vessel formation may interfere with normal development. Thus, some concerns have been raised regarding the impact of postnatal anti-VEGF treatment in preterm infants on long-term outcomes [21–23]. While anti-VEGF is used in the treatment of ROP, VEGF can be used as a biomarker with the potential to predict ROP development and monitor treatment effects [17, 24, 25].

Quantification of VEGF is associated with challenges. Studies in different research fields, i.e., cancer biology studies [26], have indicated that preanalytical procedures such as the choice of sample system and sample treatment after the blood is drawn affect the measured VEGF concentrations. In addition, Jelkmann [27] describes in a mini-review that the specificity of the assay of choice is critical for obtained VEGF levels as well as the influence of VEGF release during blood clotting. VEGF is expressed in different tissues: intracellularly [28], in the extracellular matrix [29], and in the bloodstream. In the bloodstream, VEGF is observed in both plasma and blood cells [26]. Therefore, VEGF concentrations may differ depending on the type of the sample system, e.g., serum, plasma, or whole blood, rendering the comparison of studies and application of the findings from the literature in a specific clinical setting difficult.

Even though VEGF is extensively studied in different research domains, we have not found a comprehensive systematic review covering preanalytical procedures and analytical methods for VEGF measurements. Therefore, we intended to review available evidence regarding alternative sample collection systems and the choice of analytic method for VEGF measurements. The overall aim was to evaluate the most critical factors during blood collection, sample handling, and the analytical methods that influence VEGF quantification and therefore need to be considered when planning a prospective study to get reproducible, comparable results.

## 2. Material and methods

A protocol for this systematic review was registered at PROSPERO (reference CRD42020192433) in July 2020 [30]. In addition to the review specified in the protocol, a meta-analysis of VEGF concentrations in different sample systems was included. The reporting of the results follows the Preferred Reporting Items for Systematic Review and Meta-Analysis (PRISMA) [31].

### 2.1 Article search

Pubmed and Scopus were searched according to search strategies reported in S1 Table in S1 Appendix (2021/Nov/11). The search targeted publications that compared preanalytical procedures and/or analytical methods associated with VEGF quantification. The search was performed without restrictions on language, publication date, or country. Identification of other records was performed via citation and reference searches. In addition, grey literature was searched using Google Scholar, using a few relevant search terms (“VEGF levels”, “Serum”, “Plasma”, “Measurement”, and the first 100 hits were assessed by reading titles and abstracts (2021/Oct/21)).

The searches and inclusion/exclusion process were performed by two independent researchers (U.S. and C.L.). Publications were included if they reported on how VEGF levels in blood samples were affected by preanalytical procedures, and/or sample system, and/or the analytical method. Articles were excluded if they did not meet the inclusion criteria, e.g., only reporting the comparison of biological, environmental, genetic, or physiological variations in VEGF. Conflicting views were resolved by discussion with a third reviewer (A.K.N). Rayyan QCRI was used to handle duplicates and the selection process.

### 2.2 Data extraction and quality assessment

Data were extracted using the pre-specified data extraction sheet (S2 Table in S1 Appendix). The included publications were further hand-searched to identify eligible references and citations.

The data extraction sheet includes the risk of bias evaluation according to a Swedish version of QUADAS-2 [32] and uses criteria stated by Wade et al. for bias estimation of publications in systematic reviews [33]. The risk of bias assessment covers four domains: patient selection- covering the selection of patients and if the population was representative for the review question; index test- covering the performance of the comparison (the analytical method or methods used) and the conduct and interpretation (of the analytical method and statistical comparison); reference standard; and flow and timing concerning dropouts and flow and timing in the measurements.

The extraction process was performed using the same strategy as the inclusion and exclusion process (U.S. and C.L. performed the extraction independently, and A.K.N resolved conflicts). VEGF concentrations corresponding to healthy adults were extracted if the numerical values were reported or otherwise obtained from figures using GetData Graph Digitizer version 2.26.0.20.

### 2.3 Data analysis

Concentrations of VEGF and results from statistical comparisons of analytical methods, and/or preanalytical sample handling, and/or different sample systems were extracted. The included results were extracted, and a summary is reported in S3 Table, and S1 Fig in S1 Appendix. It should be noted that none of the included publications reported a comparison of analytical methods using the same immunoassay.

The publications covering preanalytical procedures were further categorized based on the stage of the preanalytical procedure. The categorization of sample systems was based on standard abbreviations or names used for plasma systems depending on the chemical content of the anticoagulant. All types of plasma from citrate tubes were grouped as citrate-plasma (including CTAD-, and ACD-plasma), and all types of plasma from EDTA-solution tubes were grouped as EDTA-plasma (including PECT-, and Edinburgh-plasma).

Meta-analysis was used to summarize VEGF concentrations among healthy individuals, and the results were reported according to the sample systems used: whole blood, serum, citrate-plasma with in vitro activated coagulation, EDTA-, heparin- and citrate-plasma, (Table 1). The meta-analysis was used to describe and visually illustrate VEGF levels in different sample systems.

**Table 1:** Groups for meta-analysis.

Step 1: Comparing sample system	Step 2: Comparing plasma anticoagulants
Whole blood	
Serum	
Plasma	
• In-vitro activated citrate-plasma	
• Citrate-plasma	Citrate, ACD, and CTAD
• EDTA-plasma	EDTA, Edinburgh, and PECT
• Heparin-plasma	Heparin

VEGF levels in healthy adults analyzed by the same analytical method were visualized by groups in meta-analysis. In the first step, sample systems and plasma anticoagulants were compared, and in the second step, subgroups of plasma anticoagulants were compared.

The meta-package [34] in Rstudio Version 1.2.5033, R Version 4.0.4 [35] was used to create forest plots. Meta-mean was used to calculate a standardized overall untransformed mean (MRAW) with 95% confidence intervals based on each study’s reported central measure and variance. The inverse variance method was used for pooling and means and standard deviations were, when appropriate, approximated from available sample sizes, medians, ranges, and/or interquartile ranges, according to the methods proposed by Luo et al. [36] for means and those for standard deviation proposed by Wan et al. [37] and Shi et al. [38]. The R-package Forest.meta was used for illustrating the results based on a random model meta-analysis. The meta-analysis was first performed for VEGF levels in healthy individuals reported in the included publications. Then, the heterogeneity was investigated by subsetting values from the most common analytical method used, and if different healthy groups were reported in the same publication, these were summarized to one group. Further sub analyses were performed for publications using different anticoagulants for plasma: EDTA, heparin, citrate, ACD, CTAD, PECT, and Edinburgh (Table 1).

Heterogeneity was reported in the form of X^2^-test with a p-value indicating heterogeneity and the percentage variation illustrated by I^2^ statistic for all subgroups and for overall subgroup differences. P-values below 0.05 were considered significant. The meta-analysis did not include any test of publication bias.

The evidence grading was based on the following criteria: findings for each subcategory reported in more than three publications and at least 67% (2/3) indicating similar results. The quality of evidence was rated manually according to GRADE [39, 40].

## 3. Results

**Fig 1:**
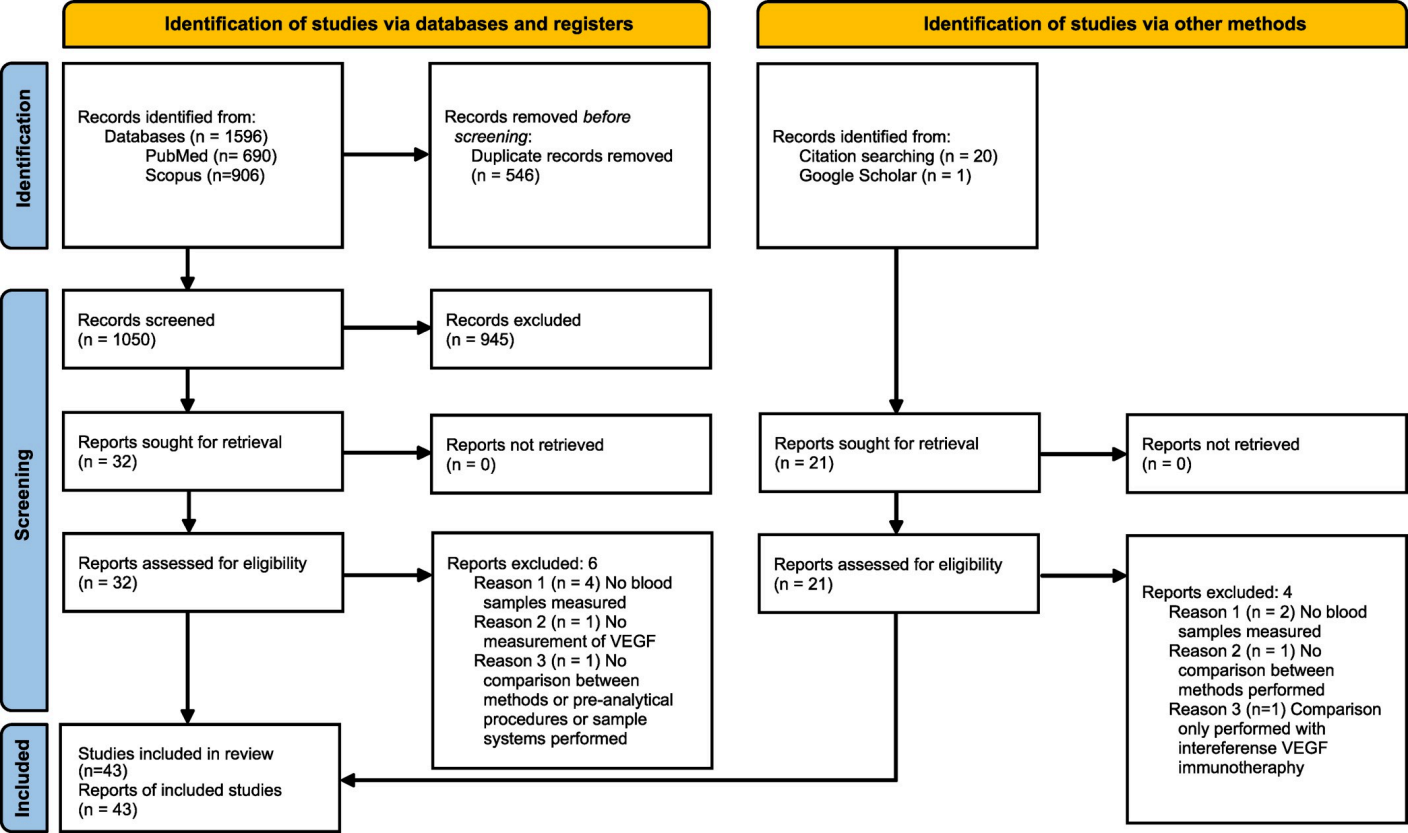
PRISMA 2020 flow diagram for new systematic review which includes searches of databases, registers and other sources. Reference: Page MJ, McKenzie JE, Bossuyt PM, Boutron I, Hoffmann TC, Mulrow CD, et al. The PRISMA 2020 statement: an updated guideline for reporting systematic reviews. BMJ 2021;372:n71.doi: 10.1136/bmj.n71. For more information, visit: http://www.prisma-statement.org/.

In total, 1596 publications were identified. After removing duplicates, 1050 publications were screened based on the title and the abstract, and 32 publications were included. Out of these 32, six publications were excluded after reading full-text (S4 Table in S1 Appendix). By examining the citations of the included publications, an additional 20 publications were identified as potentially eligible for inclusion. However, four of these were excluded based on inclusion/exclusion criteria (S4 Table in S1 Appendix). Of the first 100 hits obtained using Google Scholar, one additional publication was found (Fig 1).

Taken together, 43 studies were included in the systematic review [41–83] (Table 2). All included articles compared either preanalytical and/or methodological influences on VEGF-A concentrations. Preanalytical procedures were included in 39 studies [41–79], seven studies compared analytical methods for VEGF measurements [62, 73, 74, 80–83], and three studies reported on both preanalytical procedures and method comparisons [62, 73, 74]. The most common method used for VEGF quantification was an enzyme-linked immunosorbent assay (ELISA) from R&D (R&D Systems Inc), which was used in 30 studies [41, 43, 44, 46, 49, 50, 53–55, 57–59, 61–67, 69–78, 82] (Table 2). Eight studies used a kit for VEGF quantification based on Luminex or Bioplex platforms [42, 45, 47, 52, 56, 60, 73, 80], and 12 studies used other commercial or home-brew assays [46, 48, 51, 57, 62, 68, 74, 79–83].

**Table 2:** Overview of included publications.

Study	Study design	Funding (F) or conflict of interest(COI)	Country and year	Ethics reported Y/-	Target condition	Comparison Preanalytical (P), Methodological (M)	Method used
Adams et al. (2000)	Convenience sampling	F	U.K.	Y	Cancer	P	ELISA VEGF (R&D Systems, Abingdon UK)
Aguilar-Mahecha et al. (2012)	Convenience sampling	F & COI	Canada	Y	Sample quality	P	Luminex, Bio-PlexH Suspension Array System, and 27-plex human cytokine panel. Cat No. M50-0KCAF0Y (Bio-Rad Laboratories, Hercules, CA)
Azimi-Nezhad et al. (2012)	Convenience sampling	COI	France	Y	Sample quality	P	ELISA Quantikine human VEGF, Cat No SVE00, (R&D System, Abingdon, U.K.)
Banks et al. (1998)	Convenience sampling	F & COI	U.K.	Y	Cancer	P	ELISA, VEGF (R&D systems Europé, Abingdon, U.K.)
Biancotto et al. (2012)	Prospective	F & COI	U.S.A.	Y	Immunology	P	Luminex, using Bio-Rad Human Cytokines Group-I (27-plex) (Hercules, CA, USA)
Brookes et al. (2010)	Convenience sampling	-	U.K.	Y	Cancer	P	1. ELISA Quantikine Human–Immunoassay; (R&D Systems Europe Ltd, Abingdon, U.K.)
							2. Multiplex ELISA system (Aushon BioSystems, Boston, MA, U.S.A.) as a 4-plex comprising platelet-derived growth factor (PDGF-BB), H.G.F., FGFb, and VEGF-A.
Brøndum et al. (2016)	Prospective	COI	Denmark, 2005–2014	Y	Biomarker research	P	Luminex 100 (Bio-Plex 200 system) 8-plex panel (Bio-Plex Pro human Reagent Kit from Bio-Rad, country not specified)
Bünger et al. (2013)	Retrospective	F	Germany	Y	Colorectal cancer	P	Multiplex biochip platform, chemiluminescent sandwich immunoassay, Evidence Investigator analyzer, (Randox Laboratories Ltd. Crumlin, UK)
Dittadi et al. (2001)	Convenience sampling	F & COI	Italy	Y	Cancer	P	ELISA, Quantikine Human VEGF Immunoassay (R&D Systems, Minneapolis, USA)
Dupuy et al. (2013)	Retrospective	F & COI	France	Y	Methodological	M	1. Evidence Investigator® biochip system (Randox, Mauguio, France)
							2. Luminex, Millipore’s Multiplex Cytokine and Chemokine products xMAP ® platform. Cat no. MPXHCYTO-60K-19 (Millipore, country not specified)
George et al. (2000)	Convenience sampling	-	U.K.	-	Cancer	P	ELISA, VEGF (R&D systems, country not specified)
Ghavamipour et al. (2020)	Convenience sampling	-	Netherlands	-	Methodological	M	1. Conventional Human VEGF ELISA kit, (Abcam, Cambridge UK)
							2. Home-brew CL-ELISA
Guo et al. (2013)	Convenience sampling	F & COI	China	Y	Heart and lung disease	P	EVIDENCE 180 system (biochip), cytokine array I kits (nos. 0857 and 0658, Randox Laboratories, country not specified)
Hermann et al. (2014)	Convenience sampling	-	Germany	-	Cancer	P	Luminex,/MILLIPLEX® MAP Human Circulating Cancer Biomarker Magnetic Bead Panel (Millipore, country not specified)
Hetland et al. (2008)	Convenience sampling	F & COI	Denmark	Y	Rheumatoid arthritis	P	ELISA, Human VEGF quantitative ELISA (R&D Systems, Abingdon, Oxford, UK)
Hormbrey et al. (2002)	Convenience sampling	F & COI	U.K.	-	Sample quality	P	ELISA, Quantikine Human VEGF Immunoassay (R&D Systems, Abingdon, UK)
Kisand et al. (2011)	Prospective	F & COI	Estonia	Y	Sample quality	P	ELISA Quantikine human VEGF, Cat No. DVE00 (R&D systems Minneapolis, USA)
Krishnan et al. (2014)	Convenience sampling	F	U.S.A.	-	Sample quality	P	Luminex multiplex bead-based technology using kit from Millipore (Millipore, Billerica MA)
Kusumanto et al. (2003)	Convenience sampling	-	Netherlands	Y	Cancer	P	ELISA, Quantikine human VEGF(R&D systems, Minneapolis, USA)
							FACS measurement of cell content by MoFlo high-speed flowcytometer
Larsson et al. (2002)	Convenience sampling	F	Sweden	-	Angiogenesis	P	ELISA, Quantikine, human VEGF-A Cat No. DVE00 (R&D Systems, Minneapolis, USA)
Lee et al. (2000)	Convenience sampling	F	Korea	-	Cancer	P	ELISA, Quantikine Human VEGF Immunoassay (R&D Systems, Minneapolis, USA)
Lee et al. (2015)	Convenience sampling	COI	Korea	Y	Cancer, heart disease	P	Luminex/Milliplex M.A.P. Human Cytokine/Chemokine Magnetic Bead Panel kit- Immunology, (Millipore, country not specified)
Licht et al. (2001)	Convenience sampling	-	Germany	-	Gonadotropinstimualtion for IVF	P	ELISA Quantikine human VEGF (R&D systems Minneapolis, USA)
Lopez Yomayuza et al. (2019)	Convenience sampling	F & COI	Germany	Y	ROP	P & M	1. ELISA, VEGF R&D DuoSet (R&D systems, country not specified)
							2. AlphaLISA immunoassay (Fa. Perkin Elmer, country not specified)
Man et al. 2020	Convenience sampling	F & COI	China	Y	Cancer	M	1. Homebrew chemiluminescent assay, calibrated against VEGF165 (peprotech, country not specified), detected by VEGF165 detection probe,
							2. Human VEGF165 ELISA kit was from Miblo Co. Ltd. (Shanghai, China)
Maloney et al. (1998)	Convenience sampling	F & COI	U.S.A.	Y	Vascular diseases	P	ELISA, VEGF (R&D Systems, Minneapolis, USA)
McIlhenny et al. (2002)	Convenience sampling	-	U.K.	Y	Menstrual cycle in healthy women	P	ELISA Quantikine Human VEGF (R&D systems Europé, Abingdon, U.K.)
Ranieri et al. (2004)	Convenience sampling	-	Italy	-	Cancer	P	ELISA, Quantikine human VEGF (R&D Systems Inc., Minneapolis, USA)
Salgado et al. (2001)	Convenience sampling	F	Belgium	-	Cancer	P	ELISA, Quantikine human VEGF165 (R&D, Minneapolis, USA)
Salven et al. (1999)	Convenience sampling	F & COI	Finland, 1997	Y	Cancer	P	ELISA, Quantikine human VEGF (R&D Systems Inc., Minneapolis, USA)
Sanak et al. (2021)	Convenience sampling	COI	Switzerland	-	Eye-drops	P	Simple Plex platform (Biotechne, country not specified)
Schlingemann et al. (2013)	Convenience sampling	COI	Netherlands	Y	Diabetes type 1	P	ELISA, VEGF (R&D Systems, Abingdon, U.K.)
Starlinger et al. (2011)	Convenience sampling	-	Austria	Y	Pancreatic Cancer	P	ELISA, Quantikine Human VEGF Immunoassay (R&D Systems, Minneapolis, USA)
Svendsen et al. (2010)	Convenience sampling	COI	Denmark, 2004–2006	Y	Cancer	P	ELISA, Quantikine Human VEGF Immunoassay Cat No. DVE00, (R&D Systems, Minneapolis, USA
Verheul et al. (1997)	Convenience sampling	F	Netherlands	-	Cancer	P	ELISA VEGF (R&D Systems, Abingdon UK)
Walz et al. (2016)	Prospective	F	Germany	Y	Sample quality & methodological	P & M	ELISA Quantikine human VEGF, Cat No. DVE00 (R&D, country not specified), Luminex- Human VEGF High Sensitivity Kit, Cat No. LHSCM293 (R&D Systems, country not specified)
Webb et al. (1997)	Convenience sampling	-	U.K.	Y	Sample quality & methodological	P & M	1. R&D VEGF ELISA, mouse monoclonal anti-VEGF, R&D Systems (Abingdon, U.K.).
							2. Home-brew; capture by sflt-1 detection with in-house polyclonal anti-VEGF from rabbit against recombinant VEGF165 (Zeneca Pharmaceuticals, Alderley Edge, U.K.)
Werther et al. a) (2002)	Convenience sampling	F	Denmark	Y	Cancer	P	ELISA, Quantikine Human VEGF Immunoassay Cat No. DVE00 (R&D Systems, Minneapolis, USA)
Werther et al. b) (2002)	Convenience sampling	F	Denmark	Y	Cancer	P	ELISA, Quantikine Human VEGF Immunoassay Cat No. DVE00 (R&D Systems, Minneapolis, USA)
Wynendaele et al. (1999)	Convenience sampling	F	Belgium	Y	Cancer	P	ELISA, Quantikine human VEGF (R&D Systems Inc., Minneapolis, USA)
Yang et al. (2016)	Convenience sampling	F & COI	Taiwan	Y	Cancer	M	1. VEGF ELISA (Human VEGF165 Immunoassay, R&D systems)
							2. Homebrew- nanogold-dot-array- calibrated against Human VEGF165 PeproTech Inc. (Rocky Hill, NJ, USA)
Zamudio et al. (2013)	Prospectively collected samples	F & COI	Bolivia	Y	Pregnancy complications	P	Free VEGF, ELISA, Immunoassay kit Cat No. DVE00 (R&D Systems Minneapolis, USA)
Zhao et al. (2012)	Convenience sampling	F & COI	U.S.A.	Y	Rheumatoid arthritis	P	Mesoscale multi-spot (M.S.D.) detection. Capture ab. Peprotech, Detection ab. R&D system, Analyte standard (Peprotech, country not specified)

### 3.1 Risk of bias assessment

**Fig 2:**
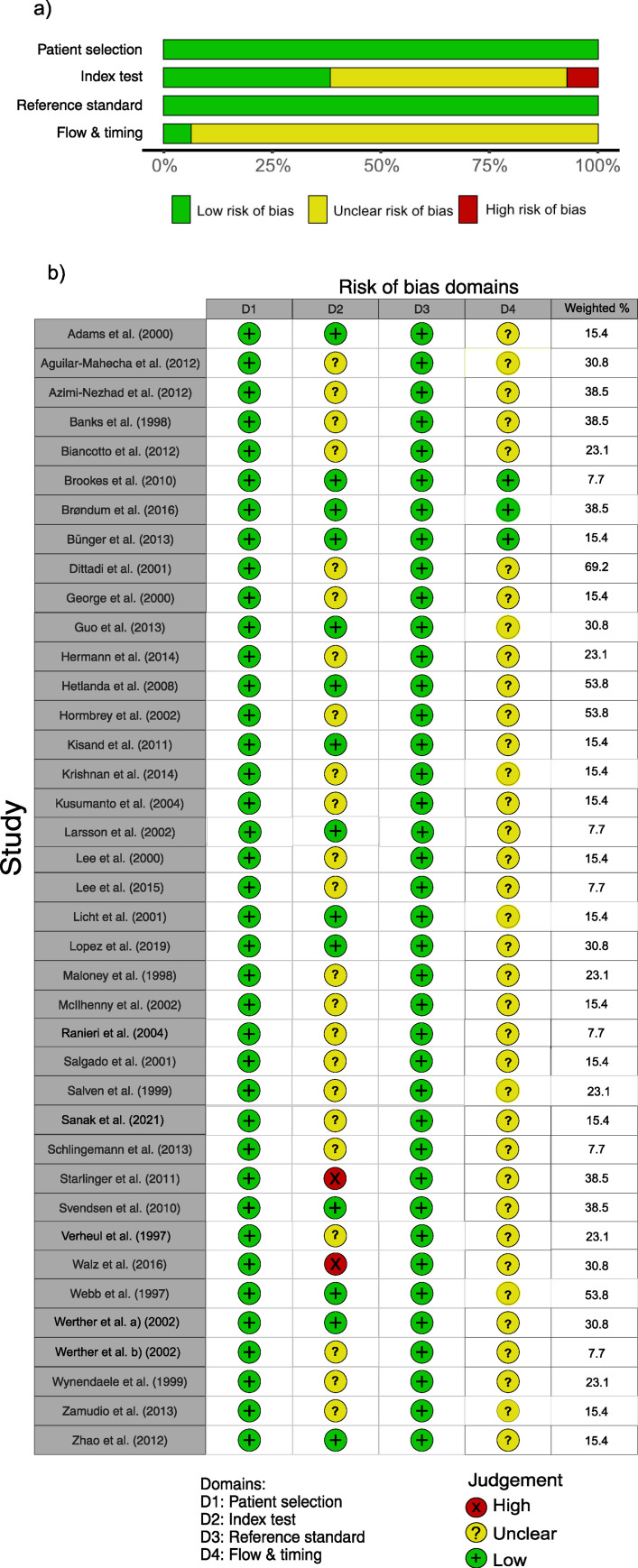
Risk of bias assessment according to the QUADAS-2 tool for diagnostic test, based on four domains. a) Summary for the 39 publications covering a comparison of preanalytical procedures. The results are weighted by the number of representative results for the 13 sub-categories included in the results. b) The risk-of-bias assessment for each of the four domains and the weighted percentage for each of the included publications.

Risk of bias assessment for individual studies was performed for publications reporting on preanalytical and/or methodological comparison. The risk of bias assessment for the 39 preanalytical studies was weighted by calculating the percentage contribution to the 13 included sub-categories in the main results. The contribution ranged between 7.7–69.2%, as shown in Table 3. The risk of bias assessment for preanalytical studies are illustrated in Fig 2. The results for the assessment of the seven included methodological publications are reported in S1 Fig in S1 Appendix.

**Table 3:** Weighted contribution of included publications related to results of preanalytical procedures.

Study	1	2	3	4	5	6	7	8	9	10	11	12	13	Total number of categories	Weighted %
Adams et al. (2000)	1					1								2	15.4
Aguilar-Mahecha et al. (2012)		1	1						1	1				4	30.8
Azimi-Nezhad et al. (2012)	1	1				1	1						1	5	38.5
Banks et al. (1998)	1	1			1	1	1							5	38.5
Biancotto et al. (2012)	1	1				1								3	23.1
Brookes et al. (2010)												1		2	7.7
Brøndum et al. (2016)	1	1				1						1	1	5	38.5
Bünger et al. (2013)												1	1	2	15.4
Dittadi et al. (2001)		1	1	1	1	1	1	1	1	1				9	69.2
George et al. (2000)			1			1								2	15.4
Guo et al. (2013)	1					1						1	1	4	30.8
Hermann et al. (2014)	1						1				1			3	23.1
Hetland et al. (2008)	1	1				1	1			1		1	1	7	53.8
Hormbrey et al. (2002)		1	1			1	1	1	1	1				7	53.8
Kisand et al. (2011)												1	1	2	15.4
Krishnan et al. (2014)			1			1								2	15.4
Kusumanto et al. (2003)				1		1								2	15.4
Larsson et al. (2002)						1								1	7.7
Lee et al. (2000)	1					1								2	15.4
Lee et al. (2015)													1	1	7.7
Licht et al. (2001)							1				1			2	15.4
Lopez Yomayuza et al. (2019)	1		1		1	1								4	30.8
Maloney et al. (1998)	1				1	1								3	23.1
McIlhenny et al. (2002)	1					1								2	15.4
Ranieri et al. (2004)						1								1	7.7
Salgado et al. (2001)			1			1								2	15.4
Salven et al. (1999)				1	1	1								3	23.1
Sanak et al. (2021)									1	1				2	15.4
Schlingemann et al. (2013)						1								1	7.7
Starlinger et al. (2011)	1	1				1	1	1						5	38.5
Svendsen et al. (2010)	1	1					1			1			1	5	38.5
Verheul et al. (1997)	1				1	1								3	23.1
Walz et al. (2016)		1				1	1				1			4	30.8
Webb et al. (1997)	1			1	1	1	1	1					1	7	53.8
Werther et al. a) (2002)				1	1	1	1							4	30.8
Werther et al. b) (2002)						1								1	7.7
Wynendaele et al. (1999)		1			1	1								3	23.1
Zamudio et al. (2013)	1		1											2	15.4
Zhao et al. (2012)	1						1							2	15.4

Description of contribution to the 13 sub-categories included in the manuscript. The weighted percentages are calculated and reported based on the number of categories each publication are represented in the manuscript.

1: Serum vs. plasma, 2: Anticoagulants used for plasma collection, 3: Fraction of samples demonstrating VEGF levels below the detection limit depending on the sample system, 4: Whole blood vs. serum or plasma, 5: Release of VEGF from stimulated platelets, 6: Meta-analysis of VEGF levels in serum, plasma, and whole blood for healthy adults, 7: Time to centrifugation, 8: Temperature before centrifugation, 9: Time for centrifugation, 10: Force, 11: Time to freezing, 12: Storage time before measurement, 13: Freeze-thaw cycles before measurement

The Quadas-2 tool is developed to assess the risk of bias in diagnostic accuracy studies and is based on four domains. The first domain of the tool, patient selection, assesses the risk of bias depending on the selection of patients and whether the included patients match the research question of the review. All included publications were assessed as low risk for bias in this domain since the comparisons were performed using the same set of samples, thus making the selection of individuals less critical. Typically, the inclusion of patients was well described; meanwhile, healthy controls were less well described, often represented by volunteers, staff, or pools of samples. Thus, 34 of 39 of the included publications were classified as using *convenience sampling* for the control group [41–44, 46, 49–72, 74–77, 79].

Regarding the second domain, the “index-test”, the risk of bias was estimated based on “concerns regarding the performance of the analytical and statistical comparison” and “the conduct or interpretation of the comparison”. In total, 14 publications were estimated as low risk [41, 46–48, 51, 53, 55, 58, 61, 62, 71, 74, 75, 79], 23 as unclear risk [42–45, 49, 50, 52, 54, 56, 57, 59, 60, 63–69, 72, 76–78], and two as a high risk of bias based on the index test [70, 73]. The risk of bias estimation for the index test is further described in S1 Appendix.

The third domain, related to reference standards assesses the risk of bias regarding “conduct and/or interpretation of the reference standard” or “concerns about the target condition”. All publications were judged as having a low risk of bias. All included publications used available commercial VEGF standards for measurements. We established contact with three providers of the most used assays in included publications (R&D Systems Inc., Merck Millipore^®^ and Randox Laboratories Ltd.), and they informed us that the used standards were calibrated in-house, and in some cases, they were also calibrated against a WHO reference with available conversion factors.

In the fourth domain, “flow and timing”, the risk of bias was estimated depending on “dropout levels from the assay” and “the flow and timing in the measurements”. Three studies gave enough information and were assessed as low risk of bias for the flow and timing domain [46–48]. The rest were assessed as unclear for this domain [41–45, 49–79]. The risk of bias estimation for the flow and timing are further described in S1 Appendix.

### 3.2 Preanalytical procedures

**Fig 3:**
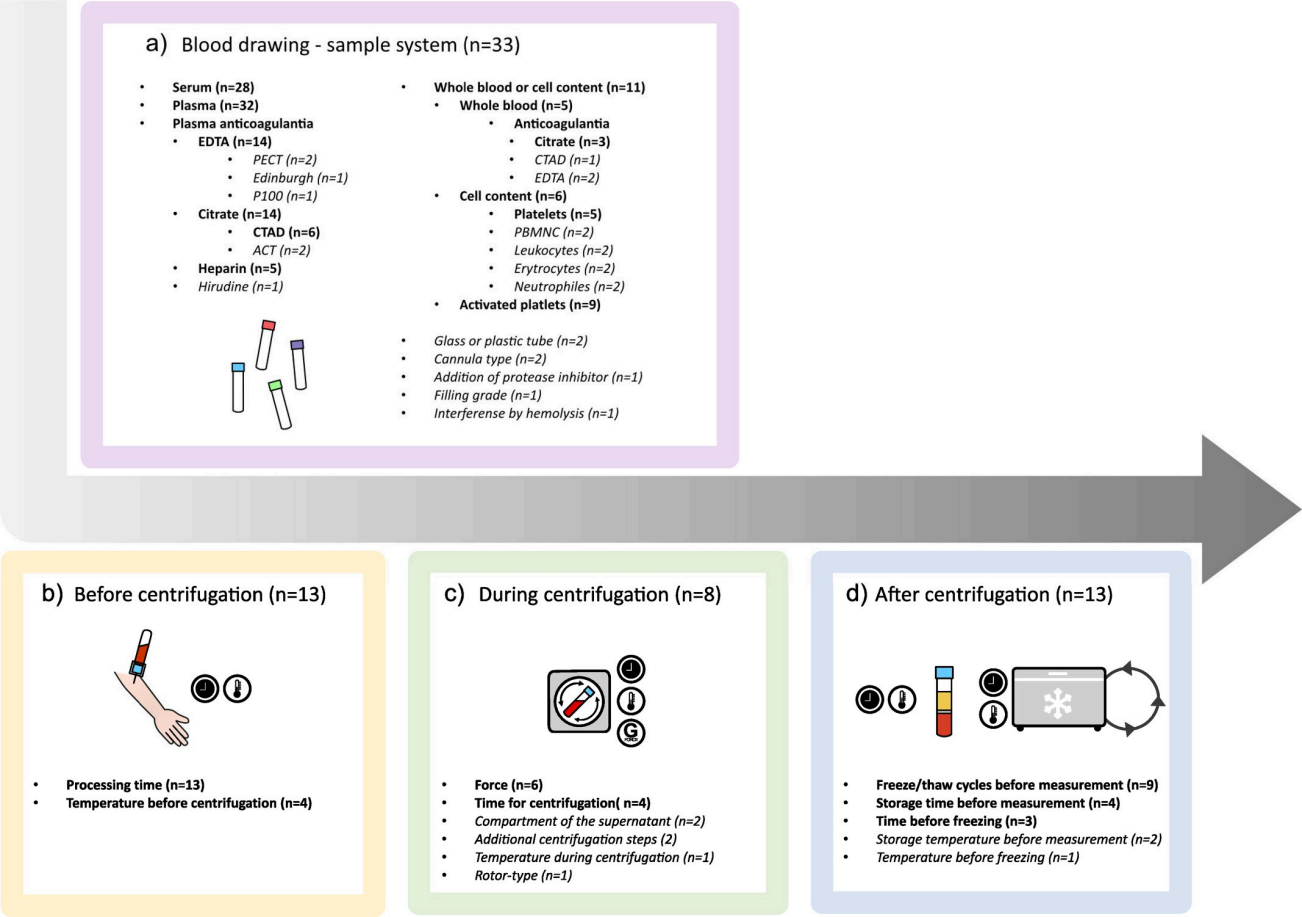
Four critical steps were identified for the preanalytical procedure presented here with the number of included publications. a) Blood drawing. b) Conditions before centrifugation. c) Conditions during centrifugation. d) Conditions after centrifugation.

The preanalytical procedures evaluated were categorized into sub-categories covering the process from drawing the blood samples to the evaluation of the time required for VEGF measurement. The blood drawing procedure was the most frequently studied aspect, along with the choice of the sample system, i.e., whole blood, serum, or plasma-anticoagulant and tubes. Four critical steps in the preanalytical process that impact VEGF measurement were identified: drawing of the blood samples and the handling before, during, and after centrifugation (Fig 3).

These four elements were further divided into sub-categories (Fig 3). For procedures investigated in less than three publications, results are reported in the S2 Appendix. A summary of findings and the quality of evidence for consistent findings are reported in Table 4.

**Table 4:** Summary of findings and evidence grading.

Finding	Critical step	Number of publications- direct comparison	Number of individuals and samples in meta-analysis	Weighted results; VEGF mean (95% CI) pg/mL	Relative effect*	Quality of evidence (GRADE)	Comments
VEGF levels are higher in serum than in plasma	Blood drawing	18	Serum; n = 906, 19 publications	Serum: 252.5 (213.1–291.9)	Serum VEGF levels were around 9 times higher than plasma VEGF levels.	Moderate ⊕⊕⊕○	-1 is based on the imprecision in VEGF concentrations
				Plasma: 27.8 (23.6–32.1)			
							-1 is based on the high probability of a different relative effect between different settings.
			Plasma; n = 1122 samples (1001 individuals), 23 publications.	17 of 18 publications comparing the systems reported higher levels in serum compared to those in plasma			
							+1 is based on the large magnitude of the effects.
VEGF is released from stimulated platelets	Blood drawing	5	Activated citrate-plasma n = 40, 4 publications	Activated citrate-plasma:156.4 (113.4–199.4)	VEGF Levels in activated citrate-plasma were around 8 times higher than those in citrate-plasma	Moderate ⊕⊕⊕○	-1 is based on the imprecision in VEGF concentrations
			Citrate-plasma n = 404, 16 publications	Citrate- plasma: 20.1 (15.8–24.4)			-1 is based on the high probability of a different relative effect between different settings
				All five publications reported increased VEGF levels after platelet activation			
							+1 is based on the large magnitude of the effects.
VEGF levels are higher in EDTA-plasma than in citrate-plasma	Blood drawing	8	EDTA-plasma; n = 697, 15 publications	EDTA-plasma: 42.1 (31.0–53.2) Citrate- plasma 20.1 (15.8–24.4)	EDTA VEGF levels were. around 2 fold higher than those in citrate-plasma levels.	Low ⊕⊕○○	-1 is based on the imprecision in VEGF concentrations
			Citrate-plasma; n = 404, 16 publications,	5 of 8 publications reported higher concentrations in EDTA-plasma compared to those in citrate-plasma			-1 is based on the high probability of a different relative effect between different settings.
Plasma has a higher fraction of samples with levels under detection limits compared to serum	Blood drawing	6	NA	Five of six publications found a higher fraction of samples with levels under the detection limit in plasma	VEGF levels cannot be analyzed since results were reported inconsistently.	Low ⊕⊕○○	-1 based on the inconsistency in how the results are reported and how they can be summarized, evaluated, and transferred
							-1 is based on the heterogeneity of the reported fractions samples under detection limits
VEGF levels are higher in heparin-plasma than in citrate-plasma	Blood drawing	3	Heparin-plasma n = 24, 3 publications	Heparin-plasma: 37.2 (32.9–41.5) Citrate- plasma 20.1 (15.8–24.4)	VEGF levels in heparin-plasma were around 2 fold higher than those in citrate-plasma.	Very Low ⊕○○○	-1 is based on the imprecision in VEGF concentrations
			Citrate-plasma n = 404, n = 16	2 of 3 publications reported higher concentrations in Heparin plasma			-1 is based on the high probability of a different relative effect between different settings.
							-1 is based on the small relative effect
VEGF levels are higher in citrate-plasma than in CTAD-plasma	Blood drawing	4	Citrate-plasma n = 294, 12 publications	Citrate- plasma 20.3 (14.8–25.9) CTAD-plasma 15.4 (10.2–20.5)	Citrate-VEGF levels were around 1.5 fold higher than CTAD-plasma VEGF levels	Very Low ⊕○○○	-1 is based on the imprecision in VEGF concentrations
			CTAD-plasma n = 117, 8 publications	3 of 4 publications reported higher concentrations in Citrate-plasma compared to those in CTAD-plasma			-1 is based on the high probability of a different relative effect between different settings.
							-1 is based on the small relative effect
VEGF levels in serum is impacted by the time to centrifugation.	Before centrifugation	10	NA	Eight of ten publications found increased VEGF levels after delayed centrifugation.	VEGF levels cannot be analyzed since results were reported inconsistently.	Low ⊕⊕○○	-1 based on the inconsistency in the results
							-1 based on the inconsistency and imprecision in how the results are reported and how they can be summarized, evaluated, and transferred
VEGF levels in EDTA-plasma is impacted by the time to centrifugation.	Before centrifugation	7	NA	Six of seven publications found increased VEGF levels after delayed centrifugation	VEGF levels cannot be analyzed since results were reported inconsistently.	Very Low ⊕○○○	-1 based on the inconsistency in the results
							-1 based on the inconsistency and imprecision in how the results are reported and how they can be summarized, evaluated and transferred
							-1 is based on the small sample size
VEGF levels in serum are stable for up to 9 freeze-thaw cycles	After centrifugation	7	NA	Five of seven publications reported no change in VEGF levels for up to 9 or 10 freeze/thaw cycles	VEGF levels cannot be analyzed since results were reported inconsistently.	Very Low ⊕○○○	-1 based on the inconsistency in the results
							-1 based on the inconsistency and imprecision in how the results are reported and how they can be summarized, evaluated, and transferred
							-1 is based on the small sample size

*The relative effect is just an approximation based on the standardized mean concentration for each sample system summarized in the meta-analysis

### 3.3 Critical step 1: Blood drawing

Of the 39 studies, 33 evaluated how VEGF concentrations depended on the tube type and the addition of anti-coagulation agents (Fig 3A), [41–45, 47, 49–54, 56–59, 62–67, 69–79]. The results are reported in detail under appropriate sub-categories. In three articles, comparing different sample systems between healthy and disease without comparing within healthy or disease, only the healthy population values were used in the meta-analysis [58, 69, 76].

#### 3.3.1 Serum vs. plasma

Eighteen studies compared VEGF concentrations between serum and plasma (S4 Table in S2 Appendix) [41, 43–45, 47, 51–53, 59, 62–64, 70–72, 74, 78, 79]. Higher levels in serum were reported in 94% (17/18 publications) of the studies [41, 43–45, 47, 51, 53, 59, 62–64, 70–72, 74, 78, 79]. One study found a difference in VEGF concentrations upon comparing serum to EDTA-plasma but not upon a comparison with heparin-plasma [47]. One study observed a difference in the concentrations upon comparing citrate and plasma but not upon comparing EDTA and heparin-plasma [44]. One study reported a non-significant difference between VEGF levels in serum and EDTA-plasma [52]. Extracted data is shared in S4 Table in S2 Appendix.

#### 3.3.2 Anticoagulants used for plasma collection

Twelve studies included a comparison between different anti-coagulation agents used for plasma collection [42–45, 47, 49, 53, 54, 70, 71, 73, 77].

EDTA- and citrate-plasma were compared in eight publications [42–44, 49, 53, 70, 71, 73]. Higher VEGF levels in EDTA-plasma were reported in 63% (5/8 publications) of the studies [43, 44, 53, 70, 73]. Banks et al. [44] reported higher VEGF levels in EDTA-plasma for one of the four included individuals; however, no difference was observed in the samples of other subjects. Starlinger et al. [70] reported moderately higher VEGF levels in EDTA-plasma than in CTAD-plasma when processed at room temperature, but no significant difference was observed at +4°C. Two studies reported no significant differences in VEGF levels between the citrate-plasma and EDTA-plasma [42, 71]. Dittadi et al. [49] found significantly lower VEGF levels in Edinburgh plasma than in citrate plasma.

EDTA and heparin-plasma were compared in three studies [45, 47, 53]. Lower VEGF in EDTA-plasma was reported in 2/3 [45, 47], and one study reported similar levels [53].

Citrate- and heparin-plasma were compared in three publications [44, 45, 53]. Lower VEGF levels in citrate-plasma were found in 2/3 publications [44, 53]. Banks et al. [44] found lower VEGF levels in citrate-plasma in one individual; however, similar levels were observed in the other three subjects. One study did not find any significant difference between systems [45].

Citrate- and CTAD-plasma were compared in four studies [49, 54, 70, 77]. Higher VEGF levels in citrate-plasma were reported in 3/4 studies [49, 70, 77]. Starlinger et al. [70] reported higher VEGF levels in citrate-plasma than in CTAD-plasma when processed at room temperature, but no difference was observed at +4°C. One study reported no significant differences in VEGF levels between the systems [54].

Extracted data is shared in S5 Table in S2 Appendix.

#### 3.3.3 Fraction of samples demonstrating VEGF levels below the detection limit depending on the sample system

The number of samples with levels below detection and/or quantification limits for different sample systems were reported in eight included publications [42, 49, 50, 54, 56, 62, 66, 78] (extracted data is shared in S4, S5 Tables in S2 Appendix). Six studies [49, 50, 56, 62, 66, 78] reported on the fraction of samples with VEGF measurements below the detection limit in plasma compared to in serum; five of those [49, 50, 62, 66, 78] observed a larger fraction of plasma samples with VEGF levels below the detection limit.

Four publications reported the fraction of undetectable VEGF levels in different plasma samples [42, 49, 54, 56]; a high proportion of undetectable samples were reported for CTAD-plasma [42, 49, 54] and Edinburgh-plasma [49]. Krishnan et al. [56] also reported undetectable levels depending on age groups, and found more samples with levels under the detection limit in the highest age group. They reported the highest frequency of detectable levels in EDTA-plasma samples (compared with serum, heparin- and citrate- plasma).

#### 3.3.4 Whole blood vs. serum or plasma

Whole blood VEGF concentrations or VEGF concentrations in different blood cells were measured in 11 publications [44, 49, 57, 62, 63, 65, 67, 72, 74, 75, 77], five of those could be compared based on a comparison between whole blood and serum or plasma, or correlation between whole blood levels and leukocyte count [49, 57, 67, 74, 75] (extracted data is shared in S6 Table in S2 Appendix). In the three studies comparing VEGF levels in whole blood with those in serum and plasma, all three found higher levels in whole blood [49, 57] and blood cells [74], respectively, compared with levels in platelet-poor plasma [57, 74] or serum [49]. VEGF levels in whole blood were correlated with leukocyte count in three publications [49, 57, 75], and granulocytes were reported as the primary source of VEGF among the leucocytes [57, 67, 75].

#### 3.3.5 Release of VEGF from stimulated platelets

Nine publications investigated the release of VEGF from platelets [44, 49, 62, 63, 67, 72, 74, 75, 77] (S6 Table in S2 Appendix). Five [44, 62, 63, 72, 74] studies compared VEGF levels before and after coagulation activation, and all showed a significant increase in VEGF levels. Eight studies correlated released VEGF with VEGF in serum, or alternatively, platelet count with VEGF in whole blood or serum, and found good agreement [44, 49, 62, 67, 72, 74, 75, 77]. However, Lopez et al. [62] did not find a correlation between platelet count and released VEGF levels among samples derived from preterm infants. Salven et al. [67] found no correlation between whole blood levels and the number of platelets in healthy subjects.

#### 3.3.6 Meta-analysis of VEGF levels in serum, plasma, and whole blood for healthy adults

The meta-analysis is based on VEGF levels from healthy adults from 28 of the 39 included publications [41, 43–45, 47, 49–51, 53, 54, 56–59, 62–67, 69, 70, 72–77]. The overall mean (95% CI) VEGF concentration in healthy adults was 55.7 (51.7–59.8) pg/mL with the meta-analysis indicating a statistically significant heterogeneity, I^2^ = 98% (S3 Appendix). The analysis found a significant difference in VEGF concentrations depending on the sample system (P<0.001). Heterogeneity ranged from I^2^ = 76% for activated citrate-plasma to I^2^ = 99%, for serum. Highest mean (95% CI) levels were found in whole blood, 390.1 (253.1–527.0) pg/mL, I^2^ = 95%, and lowest in citrate plasma, (16.7 (13.8–19.6) pg/mL, I^2^ = 95%.

**Fig 4:**
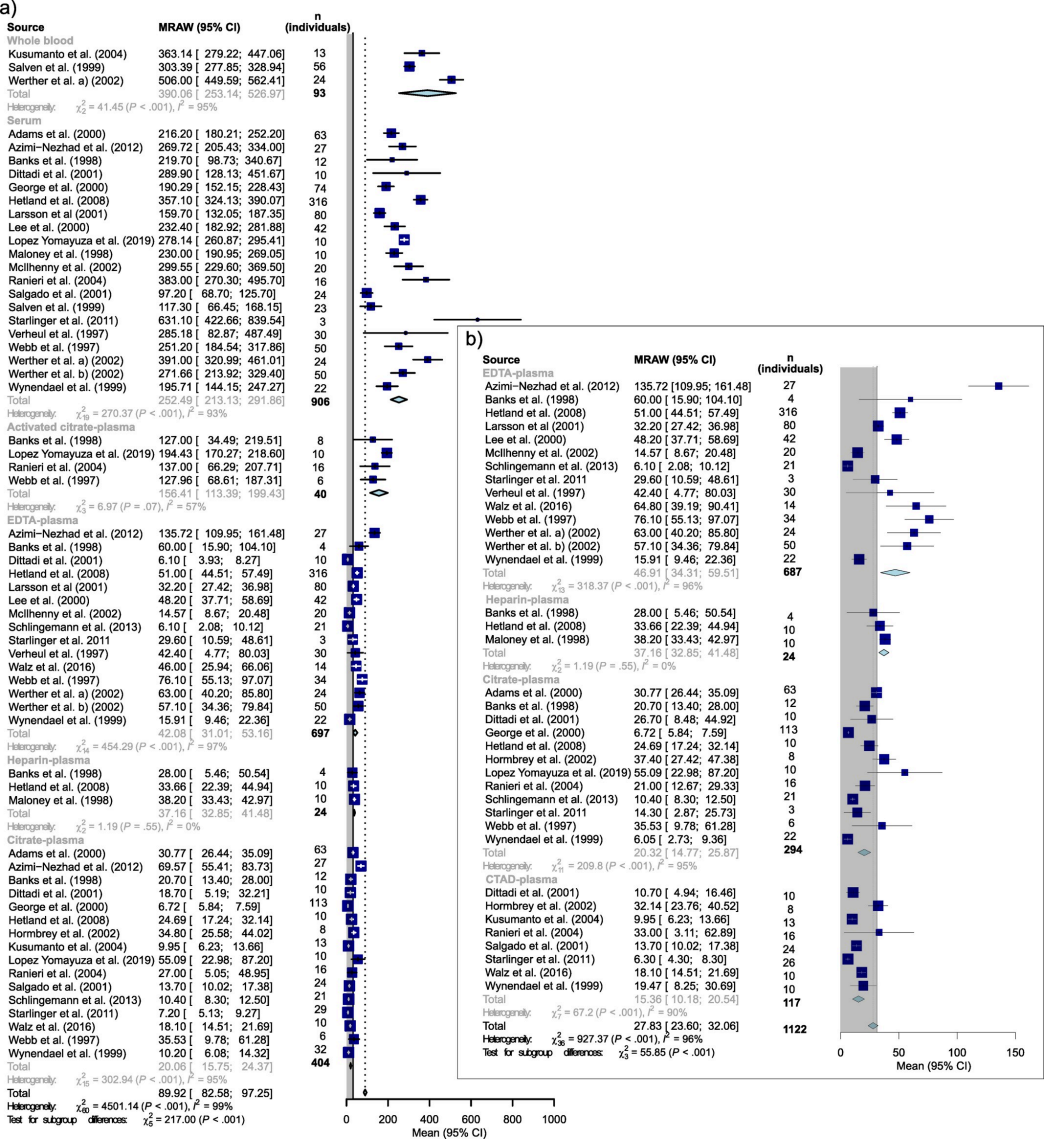
Random model meta-analysis of VEGF levels in healthy adults measured by ELISA assays from R&D Systems Inc. Measurements below the calibration range as stated by the manufacturer are shown in the grey-shaded area. Means are calculated as standardized overall untransformed mean (MRAW) with 95% confidence. a) Sub-groups based on sample system with more than three values: whole blood, serum, activated plasma, EDTA- (including PECT and Edinburgh-plasma), heparin- and citrate-plasma (including, CTAD- and ACD-plasma). **b**) Sub-analysis was performed for publications using different anticoagulants for plasma: EDTA, heparin, citrate, CTAD, ACD (not included, n = 1), Edinburgh (not included, n = 1) and PECT (not included, n = 2).

For studies using the ELISA assay from R&D to determine VEGF levels in healthy adults (24 publications), the overall mean (95% CI) was 89.9 (82.6–97.3) pg/mL, I^2^ = 99% (Fig 4A). Highest mean (95% CI) levels were found in whole blood, 390.1 (253.1–527.0) pg/mL, I^2^ = 95%, followed by serum, 252.5 (213.1–291.9) pg/mL, I^2^ = 93%, and plasma with induced coagulation or activation of platelets, 156.4 (113.4–199.4) pg/mL, I^2^ = 57%. The lowest VEGF concentrations were observed in (non-induced) plasma samples. However, the heterogeneity was statistically significant; the overall heterogeneity was I^2^ = 99%, ranging from I^2^ = 0% (n.s.) for heparin-plasma (3 publications) to I^2^ = 97% for EDTA-plasma (15 publications). Excluding the results from Starlinger et al. [70], considered as an outlier for serum values based on few included samples (n = 3) and high risk of bias, the mean (95% CI) VEGF concentration in serum changed to 243.6 (204.6–282.5) pg/mL without any change in the heterogeneity (I^2^ of 93%).

Next, VEGF concentrations in samples from different plasma types (EDTA, heparin, citrate, and CTAD) were analyzed. The mean (95% CI) VEGF level in plasma was 27.8 (23.6–32.1) pg/mL, with a statistically significant overall heterogeneity of I^2^ = 96% (ranging from I^2^ = 0% for heparin-plasma [n.s.] to I^2^ = 96% for EDTA- plasma) (Fig 4B). It is worth noting that the Quantikine R&D assay has a calibration range down to 31.3 pg/mL, i.e., higher than the mean concentration reported for plasma.

Out of the different plasma types, using EDTA as anticoagulant gave the highest VEGF concentration (mean [95% CI] 46.9 [34.3–59.5] pg/mL, I^2^ = 96%), followed by heparin (mean [95% CI] 37.2 [32.9–41.5] pg/mL, I^2^ = 0%), and citrate (mean [95% CI] 20.3 [14.8–25.9] pg/mL, I^2^ = 95%). The lowest levels were reported in CTAD-plasma (mean [95% CI] 15.4 [10.2–20.5] pg/mL, I^2^ = 90%); VEGF levels in EDTA-plasma were approximated to three-fold higher compared with CTAD-plasma. In the study by Azimi et al. [43], no units were reported for VEGF levels. Therefore, we assumed the same unit as employed in the other studies using the same method. When we instead considered their results for EDTA-plasma as outliers, the mean (95% CI) VEGF concentration for EDTA-plasma to 40.4 (28.7–52.1) pg/mL and the heterogeneity changed from I^2^ of 96% to 95%.

### 3.4 Critical step 2: Conditions before centrifugation

Thirteen studies evaluated the impact of conditions before centrifugation on VEGF levels [43, 44, 49, 52–54, 61, 70, 71, 73–75, 79] (Fig 3B and S8 Table in S2 Appendix). The time from sample acquisition to centrifugation varied from immediately after sample collection to up to a 24 h delay. The incubation before centrifugation was performed either at room temperature, +4°C, or 37°C. The temperature at which samples were stored before centrifugation was compared in four publications, [49, 54, 70, 74].

#### 3.4.1 Time to centrifugation

The time to centrifugation was evaluated in all 13 studies [43, 44, 49, 52–54, 61, 70, 71, 73–75, 79]. An increase in VEGF levels in response to longer times to centrifugation was reported in 79% (11 /13) of the studies for at least one sample system and two investigated time points [43, 44, 49, 53, 54, 61, 71, 73–75, 79]. No difference depending on the time to centrifugation was reported in two studies [52, 70]. One additional factor that might impact these results is the choice of the sample system. Nine publications examined plasma samples [43, 44, 53, 54, 61, 70, 71, 73, 74] and ten serum samples [43, 44, 49, 52–54, 70, 74, 75, 79] (S8 Table in S2 Appendix). For serum, eight studies, amounting to 80% (8/10) of the total publications [43, 44, 49, 53, 54, 74, 75, 79], reported increased levels after delayed centrifugation while two studies did not find any differences [52, 70]. The most pronounced difference in the VEGF levels in the serum was observed during the first two hours after the blood was drawn, as reported in 4/8 studies [49, 53, 74, 75]. Two publications showed an additional increase in VEGF levels after two hours, where Azimi-Nezhad reported an increase after 48 hours [43], when samples with an extra freeze-thaw cycle were included, while Hormbrey reported an increase after 6 hours [54]. Hetland et al. [53] showed a significant association with processing time up to 24 hours.

For plasma, the impact of time to centrifugation was inconsistent with concerning anticoagulants. VEGF levels in EDTA-plasma were reported to increase with the delay in six of the publications (6/7 publications examining EDTA-plasma) [43, 44, 53, 61, 71, 73], and no change was noted in one study [74]. For citrate-plasma, 3/5 studies reported a significant increase in VEGF levels depending on the time to centrifugation [43, 44, 54], while the other two studies found no difference in CTAD-plasma [70, 73]. Hormbrey et al. [54] found a significant increase in VEGF levels in citrate-plasma but not in CTAD-plasma with extended time to centrifugation.

The other included anticoagulants (hirudine and heparin) were only investigated in one publication and are presented together with results from each of the other publications reporting on how VEGF levels are affected by the time to centrifugation in S2 Appendix in S8 Table.

#### 3.4.2 Temperature before centrifugation

Of the four publications that evaluated storage temperature before centrifugation on VEGF levels, all reported changed levels depending on the temperature under one or more test conditions [49, 54, 70, 74]. Higher levels of VEGF were reported at higher storage temperatures upon comparing serum [54, 74], citrate-plasma [49, 70], and EDTA-plasma [70] samples. However, Hormbrey et al. [54] showed the opposite results for citrate-plasma with lower levels at higher temperatures, and no significant difference for CTAD-plasma. The results are presented in detail in S8 Table in S2 Appendix.

### 3.5 Critical step 3: Conditions during centrifugation

The impact of centrifugation parameters on VEGF levels was investigated in eight of the included publications [42, 46, 49, 53, 54, 68, 71, 73] (Fig 3C). Four studies compared VEGF levels depending on the centrifugation time [42, 49, 54, 68] and six depending on the centrifugal force [42, 49, 53, 54, 68, 71]. Seven studies evaluated plasma samples [42, 46, 49, 53, 54, 71, 73], and two studies investigated serum samples [53, 68]. EDTA-plasma was used in five studies [42, 46, 53, 71, 73], and citrate-plasma [49, 54] was used in two studies. Results relating to centrifugation parameters are presented in detail in S9 Table in S2 Appendix.

#### 3.5.1 Time for centrifugation

Three studies [42, 49, 54] reported a change in plasma VEGF levels depending on the centrifugation time while one found no differences for serum samples [68]. Two [42, 49] studies reported higher levels for shorter programs; these studies concurrently changed centrifugation temperature and centrifugal force when assessing the impact of time. Hormbrey et al. [54] found higher VEGF levels with longer centrifugation but only for forces above 913 *g*.

#### 3.5.2 Force

Hetland et al. [53] and Sanak et al. [68] reported no significant change in serum VEGF levels upon comparing different forces. Three studies covering centrifugal force in plasma samples reported lower VEGF levels at higher forces [53, 54, 71], and two studies showed contradictory results [42, 49].

### 3.6 Critical step 4: Conditions after centrifugation

Conditions after centrifugation, involving the time and temperature until freezing, storage time, and freeze/thaw cycles, were investigated in 13 publications [43, 46–48, 51–53, 55, 60, 61, 71, 73, 74], as shown in Fig 3D. All results relating to sample handling after centrifugation are presented in detail in S10 Table in S2 Appendix.

#### 3.6.1 Time to freezing

Three publications evaluated how the time from sample centrifugation to freezing influenced VEGF levels [52, 61, 73]. Serum was investigated in one study [52], and plasma samples were investigated in two studies [61, 73]. All studies reported increased levels of VEGF with prolonged time to freezing in at least one of the investigated sample systems and temperatures.

Maintaining serum samples at +4°C for up to 48 h between centrifugation and analysis did not significantly impact VEGF levels [52]. However, keeping serum samples at room temperature between 24 and 48 h led to an increase in VEGF levels [52]. Walz et al. [73] found that VEGF levels increased in some but not all EDTA-plasma samples after a delay of 3–6 h at +4°C until freezing; however, no change was observed in PECT-plasma samples. Licht et al. [61] reported a significant increase in VEGF levels in EDTA-plasma samples with respect to delay in centrifugation and sample storage.

#### 3.6.2 Storage time before measurement

Different durations for storage were assessed in four publications [46, 47, 51, 53]. Sample stability at -80°C (n = 2) [46, 53], -20°C (n = 1) [46], +4°C (n = 2) [46, 51] and at room temperature (n = 1) [47] was evaluated in serum (n = 2) [46, 51], and plasma (n = 4) samples [46, 47, 51, 53].

Three out of four publications reported no difference depending on the storage time [47, 51, 53]. In one study [46], eight months of storage at -80°C was associated with increased VEGF levels.

#### 3.6.3 Freeze-thaw cycles before measurement

Nine publications covered freeze-thaw cycle experiments before VEGF measurement [43, 47, 48, 51, 53, 55, 60, 71, 74]. The impact of up to ten freeze-thaw cycles was assessed on VEGF levels. Sample freezing and storage were performed at -75°C or below, and one study used liquid nitrogen [48]. The temperature used for thawing varied across studies from +4°C (on ice) up to 37°C. Included sample systems were serum (n = 7) [43, 48, 51, 53, 55, 60, 74], plasma (n = 6) [43, 47, 51, 53, 60, 71], EDTA-plasma (n = 4) [43, 53, 60, 71], heparin-plasma (n = 2) [47, 51], ACD-A plasma (n = 1) [43] and hirudin-plasma (n = 1) [43].

Five out of seven studies examining freeze-thaw cycles in serum samples [43, 51, 53, 60, 74] reported no change in VEGF levels for up to 9 [53] or 10 [43] cycles. However, Hetland et al. [53] reported significantly lower serum VEGF after ten freeze-thaw cycles compared to after 1–9 cycles. The study that used liquid nitrogen for sample storage [48] reported a trend of reduced serum VEGF with an increasing number of freeze-thaw cycles. In a study performed by Kisand et al. [55], fresh samples were compared with samples subjected to 1–3 freeze-thaw cycles. They found a significant reduction in VEGF levels after the first freeze-thaw cycle. They also reported a trend of reduction in the levels with additional freeze/thaw cycles.

Three of six publications reported altered plasma VEGF levels after freeze-thaw cycles [43, 53, 71]. Two studies noted lower VEGF levels after repeated freeze-thawing [53, 71], and one study demonstrated higher VEGF levels after repeated freeze-thaw cycles [43]. Three studies reported non-significant changes in VEGF plasma levels after up to 10 freeze-thaw cycles [47, 51, 60]. The results on the effect of freeze-thaw cycles for different plasma types are further presented in S10 Table in S2 Appendix.

## 4. Discussion

The findings here demonstrate that when analyzing circulating VEGF, the choice of sample system and the handling procedure up until centrifugation can have a major impact on the measured levels.

Even if the number of included publications in this systematic review was relatively high, it was hard to compare results between studies due to differences in study designs. For instance, some studies simultaneously changed more than one factor, hampering the possibility to deduce the effect of a single parameter. For example, the studies by performed Aguilar-Mahecha et al. [42] or Dittadi et al. [49] examined the impact of centrifugation parameters by changing durations, temperature, and force simultaneously. Another factor that rendered the comparison of the studies difficult involved differences in temperatures used during the different stages of sample handling. Several variables are known to affect immunoassay results [84, 85]. Therefore, the general advice would be to investigate the impact of these parameters one factor at a time on the analyte of choice. While the studies included in our review have compared common preanalytical steps, they may differ in other crucial study design parameters; therefore, details of the preanalytical procedure used in included publications are described in S11 Table in S2 Appendix.

Another limitation in many of the publications included in this study was that the comparison, especially for healthy subjects, was based on few samples or few pools of samples. VEGF concentrations based on healthy subjects were described but we could not compare differences between sample systems based on the extracted data since few studies reported this information comparing the same sample systems. Therefore the comparison between VEGF concentrations for different sample systems was based on description and visualization of summarized VEGF levels. Similarly, the meta-analysis showed a high degree of heterogeneity between studies, and individual study results were based on small groups of individuals with a high reported measure of variance. The heterogeneity in the meta-analysis may be attributed to the differences in preanalytical procedures used to collect the samples (S11 Table in S2 Appendix). Walz et al. [73] performed an interlaboratory comparison of VEGF levels with the exact same set of samples. They found a high degree of variability in VEGF concentrations between labs. However, although concentration differed between the laboratories, in general, a good correlation was observed. Another aspect to consider regarding the heterogeneity in the results involves the timespan between the included publications, with the first study being published in 1998 and the most recent study being published in 2021.

The risk-of-bias assessment of included publications indicates that important information was not reported for the index test and flow and timing domains. Therefore, the bias estimation could not be performed, and as a result, the quality of included results for these domains was, in most cases, unclear. The information missing in most publications included the uncertainty of the results, the experience and execution of the measurements, information about time-factors, whether the VEGF measurements were divided between analysis batches and reagent batches, and the number of samples with levels below detection and/or quantification limits.

Nevertheless, when we limited the comparison to comparable publications, we could deduce several essential factors that influenced the measured VEGF levels. These factors were categorized into four critical steps and are discussed below.

### 4.1 Critical step 1- Blood drawing

The most pronounced and described difference in VEGF levels was observed between serum and plasma samples, with approximated nine-fold higher levels in serum (moderate evidence grading). Higher levels were also reported when blood cells were not eliminated from the sample. Platelet activation results in a substantial release of VEGF (moderate evidence grading). VEGF concentrations are higher in whole blood than in serum or plasma, even if the evidence could not be evaluated for this part due to different experimental settings.

The choice of plasma anticoagulant impacts the level of platelet activation, which, in turn, influences VEGF levels. A higher number of activated platelets increases the VEGF concentration [44, 62, 63, 72, 74], and VEGF levels in serum were described to correlate with the number of platelets [49, 72, 75]. Anticoagulants added to minimize platelet activation, such as PECT and CTAD, reduce the VEGF levels compared to other anticoagulants. CTAD-plasma has lower VEGF levels compared with citrate-plasma (very low grade of evidence). The results for EDTA- and heparin-plasma were inconclusive and therefore not included in the summary of findings.

It is noteworthy that most of the measured VEGF levels in plasma, summarized in the meta-analysis using the ELISA assay derived from R&D, especially in citrate- and CTAD- plasma, were below the calibration range reported by the manufacturer. The reported fraction of samples with levels under detection limits was also higher for plasma than that for serum (low evidence grade). One study evaluated the results for levels under the quantification limit or calibration range [54] and found that concentrations below the lowest reference standard (31.3 pg/mL, R&D ELISA) were inaccurate, without linear distribution, and with higher coefficients of variation compared to concentrations above 31.3 pg/mL in other samples. The detection limit was mentioned in some of the included publications, e.g., Hetland et al. [53] used different settings to explore the detection limit. The detection limit ranged from 10.9–12.7 pg/mL, with a variation coefficient of approximately 20%. Both Adams et al. [41], McIlHenny et al. [64], and Svendsen et al. [71] reported a sensitivity of 9 pg/mL, and Larsson et al. [58] used 5 pg/mL. Zamudio et al. reported the variation coefficient to be an average (SD) of 3.3 (32.8)% in samples with levels above 5 pg/mL and 16.2 (117.6)% in samples with levels between 0 and 5 pg/mL.

The difference between the sample systems is a known problem, and this topic was also discussed or analyzed in most of the included publications. Differences in results between sample systems seem to depend on the degree of release of VEGF from intracellular compartments, also shown by five publications investigating the intracellular content of VEGF. Wartiovaara et al. [86] examined VEGF expression in peripheral blood cell fractions and found VEGF mRNA in all investigated fractions. The differences in VEGF levels and the degree of release of intracellular VEGF have also been shown by Gaudry et al. [87] under *in vitro* conditions and by Kut et al. in a meta-analysis of VEGF levels in cancer [26], and described in a mini-review by Jelkman [27].

### 4.2 Critical step 2: Before centrifugation

The time and temperature from blood drawing to centrifugation are essential to standardize when setting up a study to get reproducible results. Most studies showed an increase in VEGF levels with delayed centrifugation, while two studies reported no differences. The most pronounced difference for serum was observed during the first two hours after the drawing of the blood [49, 53, 74, 75]; we found a low grade of evidence for increased serum levels due to delayed centrifugation. The results were more inconsistent for plasma; however, citrate-and especially CTAD-plasma samples seem less sensitive to the delay than plasma samples with other anticoagulants. For EDTA-plasma, a very low grade of evidence points to increased VEGF levels with an increased delay to centrifugation.

### 4.3 Critical step 3: During centrifugation

There were few, inconsistent and contradictory results regarding the impact of centrifugation parameters, time, and force on VEGF levels. Therefore, until more evidence is available, it seems essential to standardize the centrifugation time, temperature, and force to get comparable VEGF measurements.

### 4.4 Critical step 4: After centrifugation

Results on how sample processing parameters from centrifugation to freezing, such as the temperature and time to freezing, affect VEGF levels are inconclusive. VEGF levels seem stable for several days at +4°C and months at -80°C, however, this needs further empirical evidence. Furthermore, repeated freeze-thaw cycles of serum samples seem to have little or no effect on VEGF levels (very low grade of evidence). For plasma samples, the impact of freeze-thaw cycles needs further investigation for recommendation on standardization. Half of the publications indicated no differences in VEGF levels after freeze-thaw cycles, while the other half found a significant impact of the treatment. However, this might also depend on the anticoagulants used, as different studies used different sample systems. Stable VEGF levels after freeze-thaw cycles have been shown for cerebrospinal fluid [88] and urine samples [89].

Comparing fresh samples with samples subjected to one or more freeze-thaw cycles by Kisand et al. [55] showed higher VEGF levels in fresh samples; these results were also observed for aqueous humor by Balayia et al. [90].

### 4.5. Summary

In summary, the choice of sample system is a complex issue and needs careful consideration. Recommendations have been made in some of the reviewed publications, e.g., as by Walz et al. [73]. They recommend using CTAD-plasma as they found the system to be more stable against variations in preanalytical procedures. Dittadi et al. [49] also recommend either CTAD-plasma, serum that has been allowed to clot for 2 hours, or whole blood for prospective studies depending on the blood compartment that provides the most clinically relevant information. Our analysis is consistent with the recommendations by Dittadi et al. [49]. However, the available evidence for CTAD-plasma in our systematic review is limited and the reported levels are often below the calibration range. Our findings also highlight questions regarding how to best handle serum samples, including centrifugation parameters and post centrifugation sample handling. We did not find any publications comparing methods for the preparation of whole blood samples to measure VEGF reproducibly. The results indicate that the degree of intracellular release of VEGF during the preanalytical procedure is the central part contributing to the variance in analytical measurements; this has also been shown for cerebrospinal fluid [88] and urine [89] samples.

Some publications included a comparison of VEGF levels in different sample systems relating to specific health conditions, primarily cancer. When healthy controls and patient groups were compared, the effect of different preanalytical procedures appeared comparable. In a meta-analysis, Kut et al. [26] investigated the tissue distribution of VEGF among patients with cancer. The highest levels of VEGF were found in peripheral tissues, for instance, the skeletal muscles. In a study performed by Stefanini et al. [91], the concentration of VEGF was higher in tissues than in circulating plasma. They constructed a compartment model of VEGF transport and interactions with cell receptors representing the whole human body; however, unfortunately, they did not include platelets and leukocytes in their model [91]. Ramirez et al. [92] investigated which sample system, serum, plasma, or calculated platelet content, that best reflects the humoral response to VEGF immunization of cancer patients. They found the strongest correlation between VEGF in platelets and the most pronounced humoral response.

Regarding systemic VEGF levels and the pathophysiology of ROP, the results seem contradictory. A review of 12 studies by Kandasamy et al. [93] evaluated the relationship between VEGF and ROP but could not determine whether systemic levels were related to ROP development. In addition, they did not find any evidence regarding which sample system that best reflected disease progression or systemic treatment effect of intraocular anti-VEGF injections. However, the study included a limited number of publications that used different sample systems, postnatal intervals for measuring VEGF levels, and analytical methods. Therefore, as per their recommendation, more studies should be performed to determine a mechanistic relationship between systemic VEGF levels and ROP [93].

The systemic effect on VEGF levels in premature infants after anti-VEGF treatment for ROP has been demonstrated for some available drugs [94]. Ramiréz et al. [92] found that VEGF levels in platelet were the most reliable to use when investigating the effect of VEGF-based immunotherapies. The clinical relevance of measuring platelet VEGF levels is supported by Cakir et al. [95], who found a strong association between the number of platelets, VEGF levels, and ROP in an oxygen induced retinopathy animal model and a retrospective clinical study. Korkmaz et al. [96] showed that the platelet mass index, rather than the number of platelets, was higher in infants undergoing treatment for ROP compared to in controls. They speculated that the higher platelet mass index could be related to higher VEGF content and a higher degree of stimulation of vascularization associated with platelets. We, therefore, suggest an updated in-depth systematic review of the literature to summarize available evidence regarding systemic VEGF levels and the connection to ROP pathology, including an evaluation of sample system, intervals for measurement, and analytical method.

### 4.6. Strengths and limitations

To our knowledge, this is the first systematic review and meta-analysis covering preanalytical procedures and their impact on VEGF levels that is not restricted to a specific sample system. We used a common tool for quality assessment and followed reporting guidelines according to PRISMA. The protocol was prospectively registered in PROSPERO.

This systematic review summarizes the available evidence regarding preanalytical factors important for developing protocols for sample collection to measure VEGF concentrations. We identified three main components that require special attention when designing a study where circulating VEGF is one of the outcome variables: sample system, population, and quantification method. Based on these three pillars, S2 Fig in S1 Appendix. lists aspects that need consideration for the prospective measurements of VEGF.

The main limitations of this work included the lack of analyses of publication bias for the meta-analysis. The reason for not performing an analysis of publication bias was the nature of the data, i.e., mean concentrations. We could not find any test applicable for this kind of variable. We did not conduct an in-depth search of grey literature. After consultation with a librarian at the university library, we decided not to include, for instance, unpublished lectures or clinical trials; the grey literature was limited to a search performed using Google Scholar with a limited number of keywords based on our results (VEGF levels, Serum, Plasma, Measurement). There is also a risk that relevant publications were not included since it was challenging to develop comprehensive search strategies even if much effort was taken to develop the search strategies, with a librarian’s involvement with expertise gained at the biomedical library at the university, almost half of the included publications were found by searching references and citations.

Since publications covering analytical method comparisons were limited, we could not draw any conclusions on the variability between assays, which was one of our predefined research questions. Nevertheless, we believe there is a need for a more comprehensive evaluation of consistency between available methods. R&D ELISA seems to be the most used method: 70% (30/43) of the included studies used this assay.

There is a risk of publication bias attributed to the non-reporting of negative results. Tests of preanalytical factors may likely be performed, but the results may not published if no differences are found. It is also likely that these evaluations were performed before starting a prospective collection of samples, but the results were only used internally. Methodological comparisons are routinely performed in clinical chemistry labs, nevertheless, we found a limited number of such publications.

The results from this systematic review are not helpful as a diagnostic tool. The design of this systematic review is not optimal for determining the normal range of VEGF levels in healthy adults since the inclusion of articles was based on providing a comparison between sample systems rather than including all papers measuring VEGF concentrations in healthy or diseased adults. However, a strength is that the meta-analysis clearly indicates differences in the VEGF levels based on the sample system.

The results provide vital information that should be considered when planning and designing a preanalytical protocol for sample collection in prospective studies. However, as only one study included preterm infants with ROP, a comparison of the results with respect to ROP is not possible in this setting. The difference in VEGF levels depending on the sample system was investigated in preterm infants by Lopez et al. [62] and pediatric samples by Webb et al. [74], both showing a similar pattern for VEGF levels as for adults.

## 5. Conclusion

Our results indicate that the standardization of preanalytical sample handling is necessary for reproducible and stable VEGF measurements. Our recommendation is to review the literature and consider the most critical factors, sample system, sample handling method, and analytical methods commonly used to analyze VEGF concentrations for a target condition, such as ROP, cancer, or inflammation. It is also important to report details of the study design regarding sample collection and measurements to build the evidence within a research field.

## Supporting information

S1 Checklist (DOCX)

S2 Checklist (DOCX)

S1 Protocol (PDF)

S1 Graphical abstract (TIF)

S1 Appendix (DOCX)

S2 Appendix Raw data. (XLSX)

S3 Appendix Random model meta-analysis of VEGF levels in healthy adults. (PDF)

## Abbreviations

ACD-plasma: sodium citrate, citric acid, dextrose
CTAD-plasma: sodium citrate, theophylline, adenosine, dipyridamole
EDTA-plasma: ethylenediaminetetraacetic acid
Edinburgh- plasma: EDTA, prostaglandin E1, theophylline
PECT-plasma: EDTA prostaglandin E1, Na2CO3, theophylline
