# Supplementary material for: A systematic review and meta-analysis of preanalytical factors and methodological differences influencing the measurement of circulating vascular endothelial growth factor
Source: PLoS One. 2022 Jul 6;17(7):e0270232. doi: 10.1371/journal.pone.0270232 (PMC9258884; doi:10.1371/journal.pone.0270232)
Supplement: S1 Protocol — (PDF) [file pone.0270232.s003.pdf]

Protocol for: ***Systematic review of pre-analytical factors influencing the measurement of circulating Vascular Endothelial Growth Factor (VEGF)***

Authors

Ulrika Sjöbom[1,2], ulrika.sjobom@gu.se

Anders K Nilsson[2], anders.k.nilssson@gu.se

Gunnel Hellgren[2,3], gunnel.hellgren@gu.se

Hanna Gyllensten[1,4], hanna.gyllensten@gu.se

Ann Hellström[2], ann.hellstrom@medfak.gu.se

Chatarina Löfqvist[1,2], chatarina.lofqvist@gu.se

[1]Institute of Health and Care Science, Sahlgrenska Academy, University of Gothenburg, Box 457, SE-405 30 Gothenburg, Sweden

[2]Department of Neuroscience, Institution of Neuroscience and Physiology, Sahlgrenska Academy, University of Gothenburg, Gothenburg, Sweden

[3]Institute of Biomedicine, Sahlgrenska Academy, University of Gothenburg, Gothenburg, Sweden

[4]Centre for Person-Centred Care (GPCC), University of Gothenburg, Box 457, SE-405 30 Gothenburg, Sweden.

Contact, corresponding author

Ulrika Sjöbom, Institute of Health and Care Science, Sahlgrenska Academy, University of Gothenburg, Box 457, SE-405 30 Gothenburg, Sweden, E-mail ulrika.sjobom@gu.se, Phone: +46709-995166

Contributions

US and CL designed the database search and data extraction. AKN, GH, HG and AH Contributed regarding expertise on clinical aspects, pre-analytical factors and analytical methods. US drafted the protocol and all authors read and approved the final protocol.

Financial Support

US, AKN, GH, HG, AH and CL was financed by De Blindas Vänner. AH was financed bythe Swedish Research Council (#2015-00810, KBF-3 and #2016-01131), Gothenburg Medical Society Governments grants under the ALF-agreement (ALFGBG-717971 and ALFGBG-812951) and Wallenberg Clinical Scholars.

Introduction- rationale of the review

The survival rate for babies born extremely premature has increased significantly during the last decades (Fellman, Hellström-Westas et al. 2009, Serenius, Källén et al. 2013). Premature birth is associated with several morbidities that are connected to the immaturity. One morbidity only affecting premature babies is retinopathy of prematurity (ROP). In ROP, the vascularisation of the retina after birth undergoes pathological changes with risk for detachment of the retina if not treated (Hellström, Smith et al. 2013). Early stages of the disease can only be detected by repeated eye examinations which can be stressful for the babies. If ROP progresses to severe stages, treatment is needed with the intention to inhibit the vascularisation process by laser or intra-ocular injections of molecules targeting the vascularisation process (Blencowe, Moxon et al. 2016). It would be preferable to minimize the number of eye-examinations since the disease only progress to severe ROP that requires treatment in a minor part of the infants. There are several known risk factors for severe ROP, such as low birth weight and poor postnatal growth, which can be used as predictors of disease development. There are also some known growth factors connected to the pathology of ROP, where Vascular Endothelial Growth Factor (VEGF) have a great importance for vessel growth (Hellström, Smith et al. 2013).

VEGF is a growth hormone belonging to the PDGF-superfamily. VEGF proteins are expressed from four different genes in four different variants: VEGF- A, B, C and D (Holmes and Zachary 2005). VEGF-A is the most studied of these proteins and is the protein referred to if not otherwise specified. Each of the proteins are also expressed in different splice-variants, i.e. isoforms (Woolard, Bevan et al. 2009). VEGF-A has been extensively studied because of its importance for vessel growth in tumours (Dvorak, Brown et al. 1995). VEGF-A is also important for embryonic development (Dumont, Fong et al. 1995) and this protein has connection to the pathogenesis behind preeclampsia (Phipps, Thadhani et al. 2019) why it also is well studied in pregnancy.

VEGF-A has been identified as a possible target for treatment of retinopathy via intraocular injection of anti-VEGF molecules (Ferrara, Gerber et al. 2003). The injection of anti-VEGF drugs reduce new blood vessel growth (neovascularisation) or oedema (swelling) (Li, Busoy et al. 2018). Anti-VEGF drugs may be used to treat a number of eye conditions that cause new blood vessel growth or swelling under the macular area of your retina, at the back of the eyes. Currently, in adults anti-VEGF drugs are used in the treatment of wet age-related macular degeneration (wet AMD), diabetic macular oedema (DMO), macular oedema caused by retinal vein occlusion, and myopic choroidal neovascularisation (myopic CNV) (Freund, Korobelnik et al. 2015). Recently, intraocular anti-VEGF treatment has become an alternative to laser treatment for infants with severe ROP (Cayabyab and Ramanathan 2016).

To investigate the effect of intraocular anti-VEGF treatment in neonates, levels of VEGF-A and anti-VEGF has been determined in the blood after treatment (Stahl, Krohne et al. 2018). Since VEGF has important functions during development in several organ systems like the brain, the lungs, the kidneys and the gut (Dumont, Fong et al. 1995, Ferrara, Gerber et al. 2003). It is important to consider whether the infant’s normal general vascular development is affected by the treatment. Recently, possible negative effects on other organ systems have been published (Morin, Luu et al. 2016, Kunzmann, Ngyuen et al. 2019). The different available studies about VEGF after anti-VEGF treatment in infants with ROP have been performed using diverse sample system and methods for the measurement of VEGF (Walz, Boehringer et al. 2016). It is known that VEGF levels are highly variable between different sample systems and that different analysis methods are targeted at specific structures of the VEGF molecule with diverse specificity. Inconsistent results between

studies can be explained by the dispersion due to different methods used for analysis. Moreover, it also seems like the results to a high degree can be affected by the pre-analytical handling of the samples (Walz, Boehringer et al. 2016).

When using VEGF gene products or isoforms for prediction of disease or monitoring the systemic effect of anti-VEGF treatment, it is important with well validated and reliable methods for the quantification. Therefore, we aim to systematically review how the method of choice for VEGF quantification affects the obtained results. We also aim to systematically review how pre-analytical handling and choice of sample system affect the VEGF concentration.

Analytical/ Immunoassays variability

Even a robust immunoassay can be affected by analytical errors such as cross-reacting substances, anti-analyte antibodies or anti-reagent antibodies (Sturgeon and Viljoen 2011). It is also important to remember that different immunoassays for the same analyte are based on different antibodies, mono- or polyclonal. Different antibodies have different specificity regarding epitope and different affinity for the antigen (Polacchini, Metelli et al. 2015). The calibration can also differ between different assays and contribute to discrepancy between assays (Whicher 1991). This means that available commercial assays for measurement of VEGF differ in the specificity for VEGF gene-variants and splicing- isoforms as well as they have a diversity in cross-reactivity with closely related molecules.

Pre-analytical

The pre-analytical phase is defined in the international standard for medical laboratory (SS-EN, ISO 15189:2012): “as all steps from the clinician's request of a test to the analytical examination starts”. The pre-analytical phase is the phase with the highest contribution to analytical errors, approximately 46-68 % of the total error at the medical laboratory (Plebani 2006). Sometimes the pre-analytical phase can be divided into a pre-pre analytical phase which refers to everything affecting the results that are out of control for the laboratory personal (Plebani 2006). The true pre-analytical phase includes everything from receiving the samples at the laboratory until the examination starts. In the medical laboratory, those steps are steadily monitored and evaluated by quality assurance programs investigating the frequency and importance of errors defined by quality indicators (Plebani, Sciacovelli et al. 2015). When biomarkers are used in studies during drug-development, there are recommendations for specimen preservation and stability assessments (Dakappagari, Zhang et al. 2017). Factors that contributes to biomarker stability has been divided in:

- Inter- and intra-individual factors such as biological factors, genetic makeup and environmental and physiological factors (Dakappagari, Zhang et al. 2017)
- Pre-analytical factors such as sample collection/storage at clinical site, sample shipment and transport, sample handling and storage at bioanalytical facility (Dakappagari, Zhang et al. 2017).

Research question

The question for the review is how the measured VEGF concentration is affected by the choice of method for measurement. The reason is to summarize available comparisons between commercial available methods. We also aim to investigate how pre-analytical factors influence the measurement of VEGF. The review will cover all available VEGF gene-products and isoforms. The results will be important, if VEGF would be used for the prediction of ROP or if circulating VEGF levels would be followed after intraocular injection of anti-VEGF drug, to understand the clinical relevance in

obtained results. The rationale is to summarize existing information to be easily available and to help design and implement future studies in the field. Since biological variability is fixed, this review will be limited to the pre-analytical factors beginning with the sampling procedure. Collected information about how the choice of method and which pre-analytical factors that affect the measurement of VEGF could be useful for the decision of sampling protocol and method of choice for upcoming studies, it will also gather valuable information about if there are information gaps in this field that needs to be filled. Using harmonized protocols for the sampling procedure is valuable for the possibility to compare results between studies.

Methods

Eligibility criteria- Studies will be selected according to following criteria:

Studies included have to cover measurement of any VEGF gene product with any commercial available immunoassay independent of isoform specificity and some kind of evaluation about how methodological and pre-analytical factors influence the results. Studies will be excluded if they cover biological, environmental, genetic or physiological variations or pre-analytical factors out of control such as misidentification of patients. All possible populations and study designs will be included if they are a part of a VEGF methodological evaluation. To be considered for inclusion, the study needs to include some information on how VEGF concentration is affected by methodological differences and pre-analytical. The study design must include a systematic comparison between methodological differences and/or pre-analytical handling performed using at least two groups of comparable samples measured or treated in different ways. No secondary outcomes are will be considered in this review.

Categorisation of results

The results will be categorised based on the specificity of the used method and type of comparison, pre-analytical handling or methodological comparison.

Different pre-analytical steps will be categorised based on:

- Sample system
    - Serum
    - Plasma- use of anticoagulant
    - Whole blood
    - Others
- Methodology for sampling
    - Venous
    - Arterial
    - Type and size of needle
    - Location of puncture
    - Delay to centrifugation
    - Temperature
- Centrifugation
    - Time
    - Speed
    - Angle
    - Temperature
- Transportation conditions
- Storage conditions

  - Temperature
  - Freeze/Thaw cycles
  - Time
- Sample interferences
  - Haemolysed
  - Icteric
  - Lipemic samples

Information source:

Search strategy: Search terms are developed and reviewed by a librarian with expertise on search strategy for systematic reviews at the biomedical library, University of Gothenburg. The search will be performed using English without restriction to dates of publications, status of publication or language of the results. Databases that will be used for this review are PubMed and Scopus and the search terms are presented below. To investigate the possibility that articles of interest have been missed in the database searches, we will check the reference lists in included articles to look for additional relevant publications.

Scopus: TITLE-ABS-KEY ("Vascular Endothelial Growth Factor A" OR vegf OR vegf-a OR "Vascular Endothelial Growth Factor" OR "Vascular Permeability Factor") AND TITLE-ABS-KEY (preanalytical OR pre-analytical OR pre-analyze OR preanalyze OR handling OR comparison* OR measurement* OR validation OR standardization OR "blood specimen" OR "specimen handling" OR "blood collection" OR "blood samples" OR "blood sample" OR "platelet activation" OR "platelet stimulation" OR "platelet inhibitor" OR "mechanical stimulation" OR dilution) AND TITLE-ABS-KEY (anticoagulants OR anticoagulation OR anticoagulant OR cryopreservation OR freezing OR temperature OR "time factors" OR centrifugation OR centrifuge OR thawing OR storage)

PubMed: (Vascular Endothelial Growth Factor A[mesh] OR vascular Endothelial Growth Factor A[tiab] OR VEGF[tiab] OR VEGF-A[tiab] OR Vascular Endothelial Growth Factor[tiab] OR Vascular Permeability Factor[tiab]) AND (preanalytical[tiab] OR pre-analytical[tiab] OR pre-analyze[tiab] OR preanalyze[tiab] OR handling[tiab] OR comparison[tiab] OR comparisons[tiab] OR measurement[tiab] OR measurements[tiab] OR validation[tiab] OR standardization[tiab] OR blood specimen collection[mesh] OR blood specimen[tiab] OR specimen handling[mesh] OR specimen handling[tiab] OR blood collection[tiab] OR blood samples[tiab] OR blood sample[tiab] OR platelet activation[tiab] OR platelet stimulation[tiab] OR platelet inhibitor[tiab] OR mechanical stimulation[tiab] OR dilution[tiab]) AND (Anticoagulants[mesh] OR anticoagulation[tiab] OR anticoagulants[tiab] OR anticoagulant[tiab] OR Cryopreservation[mesh] OR cryopreservation[tiab] OR Freezing[mesh] OR freezing[tiab] OR Temperature[mesh] OR temperature[tiab] OR Time Factors[mesh] OR time factors[tiab] OR centrifugation[mesh] OR centrifugation[tiab] OR centrifuge[tiab] OR thawing[tiab] OR storage[tiab])

Study records

Selection process

The selection process first part will be performed by two independent (US and CL) reviewers who will conduct the search and screen for articles according to eligibility criteria based on title and article abstract. In the second step, included articles will be requested in full-text and inclusion will be discussed further, a third part (AKN) will be involved to resolve any disagreements in the first round of literature selection. The process for inclusion and exclusion will be described in a “PRISMA”-flow chart with reason for exclusion.

Data collection

The data collection will start 15 august 2020 and the review is expected to be completed 15 December 2020. The results from Scopus and Pubmed will be imported to Rayyan QCRI which will be the software used to remove duplicate publications and the tool to handle the inclusion and exclusion procedure. Information from included articles will be collected according to a data extraction sheet with variables defined in appendix A. The data extraction sheet will be piloted for 10 papers and if necessary updated before the review process starts. If results are missing the corresponding author will be contacted for additional information. If information about specificity of used assay or assays are incomplete the marketing company will be contacted.

Strategy for data synthesis

Data will be presented and summarized to create an overview of available information about methodological differences when measuring VEGF and which pre-analytical factors that influence the results. The data will not be used for any statistical calculations.

Results will be presented for subgroups, as defined under "Categorisation of results", to be able to compare results between similar investigations. The comparison will be based on the homology in the results and will cover the strength and weaknesses in those.

Quality of evidence, risk of bias

The quality of evidence will be assessed according to the Swedish version of Quadas-2 (SBU), using criteria stated in Appendix B (Wade, Corbett et al. 2013). Criteria for evaluation of Bias is a modified version of Quadas-2 adjusted to be suitable for bias estimation during a systematic review. The reproducibility or coefficient of variation can be used to estimate the importance of variation in concentrations between methods or different treatment strategies of samples. The design of the test, the number and type of samples, the occurrence of incomplete data, the risk for selective reporting and funding or conflicts of interest will be evaluated to estimate the reliability of the results and risk of bias. This information will be summarized as degrees of reliability of the results, low, high or unclear risk. The judgement will be performed un-blinded by two independent reviewers (US and CL) and the third part (AKN) will do the final judgement. The degree of reliability will be presented together with the results in the report. There is an overall risk of bias according to reporting of those results since there might be a risk methodological or treatment comparison is not reported if no differences were found.

Additional information:

PRISMA-P checklist 2015 was followed for this protocol according to the Explanation and Elaboration article (Liberati, Altman et al. 2009).

Appendix A; Data extraction sheet

The following items will be collected from included papers:

- Aim of the study
- Study design
- Funding or conflict of interest
- Number of participants and descriptions of participants
- Time for study
- Country and place for the study
- Assay or assays for measurement
- The specificity of the assay- will be collected from the company that market the assay if not mentioned in the article
- Reproducibility for the measurements: intra- and or inter assay coefficient of variation
- Parameters for comparison
- Number and type of samples for used
- Concentrations of VEGF for different methods and treatment

Appenidx B; criteria for evaluation of methodological quality

Earlier published "*Quality assessment of comparative diagnostic accuracy studies: our experience using a modified version of the QUADAS-2 tool"* (Wade, Corbett et al. 2013).

The following items will be assessed in included papers based on four domains 1. Patient selection, 2. Index test, 3. Reference standard and 4. Flow and timing

1. If diagnostic randomised controlled trials are included in the review, were appropriate methods used to generate the allocation sequence and conceal the allocation of tests?

Domain 1

Consider whether the methods used may have resulted in biased patient selection. Both randomisation method details and result details can be used to assess risk of bias. If, at baseline, important prognostic factors are balanced across groups, patient selection can be judged as being at low risk of bias (even if the method of reporting is unclear).

2. Were the index test results interpreted without knowledge of the results of the comparator test (and vice versa)?

Domain 2

Were blinding or randomisation methods used? If not, were they needed? Did the individual tests require objective or subjective interpretation?

3. Were investigators appropriately experienced (established tests) and trained (new tests) to adequately perform both tests?

Domain 2

Accreditation, number of years of experience and number of procedures/tests performed. Time spent on training. Manufacturer guidance on training.

4. Was the execution of both tests as they should be performed in clinical practice?

Domain 2

Manufacturer guidance on appropriate test use. Atypical methods/procedures used? Current good practice guidance used?

5. Were the index and the comparator tests independent?

Domain 2

Did one test form part of another?

6. Were the reference standard and the index/comparator tests independent of each other?

Domain 3

Did the index or comparator test form part of the reference standard?

7. Were the results of both tests verified using the same reference standard?

Domain 4

The reference standard used may vary depending on individual patient test results. For example, some may have biopsy or further tests, whereas others may just have a follow-up visit at a much later date.

8. Was there an appropriate interval between the index and the comparator tests?

Domain 4

Rate of disease development. Possibility of disease status change (improvement or deterioration).

9. Did the whole sample undergo both tests (or one test, if study was randomised)?

Domain 4

Consider whether differences in test characteristics might result in differences in drop-out rates.

10. Was there a difference in the number of uninterpretable or indeterminate results between tests, which is likely to have biased the study results?

Domain 4

Reasons for such results could be related to test or patient characteristics (possible bias issue) or could be due to chance, for example, equipment failure, which may be common when using developing technology (although this will reduce study power, it is unlikely to be an important bias issue).

References

Blencowe, H., S. Moxon and C. Gilbert (2016). "Update on Blindness Due to Retinopathy of Prematurity Globally and in India." Indian Pediatr **53 Suppl 2**: S89-s92.

Cayabyab, R. and R. Ramanathan (2016). "Retinopathy of Prematurity: Therapeutic Strategies Based on Pathophysiology." Neonatology **109**(4): 369-376.

Dakappagari, N., H. Zhang, L. Stephen, L. Amaravadi and M. U. Khan (2017). "Recommendations for clinical biomarker specimen preservation and stability assessments." Bioanalysis **9**(8): 643-653.

Dumont, D. J., G. H. Fong, M. C. Puri, G. Gradwohl, K. Alitalo and M. L. Breitman (1995). "Vascularization of the mouse embryo: a study of flk-1, tek, tie, and vascular endothelial growth factor expression during development." Dev Dyn **203**(1): 80-92.

Dvorak, H. F., L. F. Brown, M. Detmar and A. M. Dvorak (1995). "Vascular permeability factor/vascular endothelial growth factor, microvascular hyperpermeability, and angiogenesis." Am J Pathol **146**(5): 1029-1039.

Fellman, V., L. Hellström-Westas, M. Norman, M. Westgren, K. Källén, H. Lagercrantz, K. Marsál, F. Serenius and M. Wennergren (2009). "One-year survival of extremely preterm infants after active perinatal care in Sweden." Jama **301**(21): 2225-2233.

Ferrara, N., H. P. Gerber and J. LeCouter (2003). "The biology of VEGF and its receptors." Nat Med **9**(6): 669-676.

Freund, K. B., J. F. Korobelnik, R. Devenyi, C. Framme, J. Galic, E. Herbert, H. Hoerauf, P. Lanzetta, S. Michels, P. Mitchell, J. Monés, C. Regillo, R. Tadayoni, J. Talks and S. Wolf (2015). "TREAT-AND-EXTEND REGIMENS WITH ANTI-VEGF AGENTS IN RETINAL DISEASES: A Literature Review and Consensus Recommendations." Retina **35**(8): 1489-1506.

Hellström, A., L. E. H. Smith and O. Dammann (2013). "Retinopathy of prematurity." Lancet (London, England) **382**(9902): 1445-1457.

Holmes, D. I. R. and I. Zachary (2005). "The vascular endothelial growth factor (VEGF) family: angiogenic factors in health and disease." Genome biology **6**(2): 209-209.

Kunzmann, S., T. Ngyuen, A. Stahl, J. M. Walz, M. M. Nentwich, C. P. Speer and K. Ruf (2019). "Necrotizing enterocolitis after intravitreal bevacizumab in an infant with Incontinentia Pigmenti - a case report." BMC Pediatr **19**(1): 353.

Li, Y., J. M. Busoy, B. A. A. Zaman, Q. S. W. Tan, G. S. W. Tan, V. A. Barathi, N. Cheung, J. J.-Y. Wei, W. Hunziker, W. Hong, T. Y. Wong and C. M. G. Cheung (2018). "A novel model of persistent retinal neovascularization for the development of sustained anti-VEGF therapies." Experimental Eye Research **174**: 98-106.

Liberati, A., D. G. Altman, J. Tetzlaff, C. Mulrow, P. C. Gøtzsche, J. P. A. Ioannidis, M. Clarke, P. J. Devereaux, J. Kleijnen and D. Moher (2009). "The PRISMA statement for reporting systematic reviews and meta-analyses of studies that evaluate health care interventions: explanation and elaboration." PLoS medicine **6**(7): e1000100-e1000100.

Morin, J., T. M. Luu, R. Superstein, L. H. Ospina, F. Lefebvre, M. N. Simard, V. Shah, P. S. Shah and E. N. Kelly (2016). "Neurodevelopmental Outcomes Following Bevacizumab Injections for Retinopathy of Prematurity." Pediatrics **137**(4).

Phipps, E. A., R. Thadhani, T. Benzing and S. A. Karumanchi (2019). "Pre-eclampsia: pathogenesis, novel diagnostics and therapies." Nature reviews. Nephrology **15**(5): 275-289.

Plebani, M. (2006). "Errors in clinical laboratories or errors in laboratory medicine?" Clin Chem Lab Med **44**(6): 750-759.

Plebani, M., L. Sciacovelli, A. Aita, M. Pelloso and M. L. Chiozza (2015). "Performance criteria and quality indicators for the pre-analytical phase." Clin Chem Lab Med **53**(6): 943-948.

Polacchini, A., G. Metelli, R. Francavilla, G. Baj, M. Florean, L. G. Mascaretti and E. Tongiorgi (2015). "A method for reproducible measurements of serum BDNF: comparison of the performance of six commercial assays." Scientific Reports **5**(1): 17989.

SBU. (2019-10-19). Retrieved 2020-06-17, 2020, from https://www.sbu.se/globalassets/ebm/quadas-2.pdf.

Serenius, F., K. Källén, M. Blennow, U. Ewald, V. Fellman, G. Holmström, E. Lindberg, P. Lundqvist, K. Maršál, M. Norman, E. Olhager, L. Stigson, K. Stjernqvist, B. Vollmer and B. Strömberg (2013). "Neurodevelopmental outcome in extremely preterm infants at 2.5 years after active perinatal care in Sweden." Jama **309**(17): 1810-1820.
Stahl, A., T. U. Krohne, N. Eter, I. Oberacher-Velten, R. Guthoff, S. Meltendorf, O. Ehrt, S. Aisenbrey, J. Roider, H. Gerding, C. Jandeck, L. E. H. Smith, J. M. Walz, S. Comparing Alternative Ranibizumab Dosages for and G. Efficacy in Retinopathy of Prematurity Study (2018). "Comparing Alternative Ranibizumab Dosages for Safety and Efficacy in Retinopathy of Prematurity: A Randomized Clinical Trial." JAMA pediatrics **172**(3): 278-286.
Sturgeon, C. M. and A. Viljoen (2011). "Analytical error and interference in immunoassay: minimizing risk." Ann Clin Biochem **48**(Pt 5): 418-432.
Wade, R., M. Corbett and A. Eastwood (2013). "Quality assessment of comparative diagnostic accuracy studies: our experience using a modified version of the QUADAS-2 tool." **4**(3): 280-286.
Walz, J. M., D. Boehringer, H. L. Deissler, L. Faerber, J. C. Goepfert, P. Heiduschka, S. M. Kleeberger, A. Klettner, T. U. Krohne, N. Schneiderhan-Marra, F. Ziemssen and A. Stahl (2016). "Pre-Analytical Parameters Affecting Vascular Endothelial Growth Factor Measurement in Plasma: Identifying Confounders." PLoS One **11**(1): e0145375.
Whicher, J. T. (1991). "Calibration is the key to immunoassay but the ideal calibrator is unattainable." Scand J Clin Lab Invest Suppl **205**: 21-32.
Woolard, J., H. S. Bevan, S. J. Harper and D. O. Bates (2009). "Molecular diversity of VEGF-A as a regulator of its biological activity." Microcirculation (New York, N.Y. : 1994) **16**(7): 572-592.
