# Supplementary figures and images for: A systematic review and meta-analysis of preanalytical factors and methodological differences influencing the measurement of circulating vascular endothelial growth factor
Source: PLoS One. 2022 Jul 6;17(7):e0270232. doi: 10.1371/journal.pone.0270232 (PMC9258884; doi:10.1371/journal.pone.0270232)

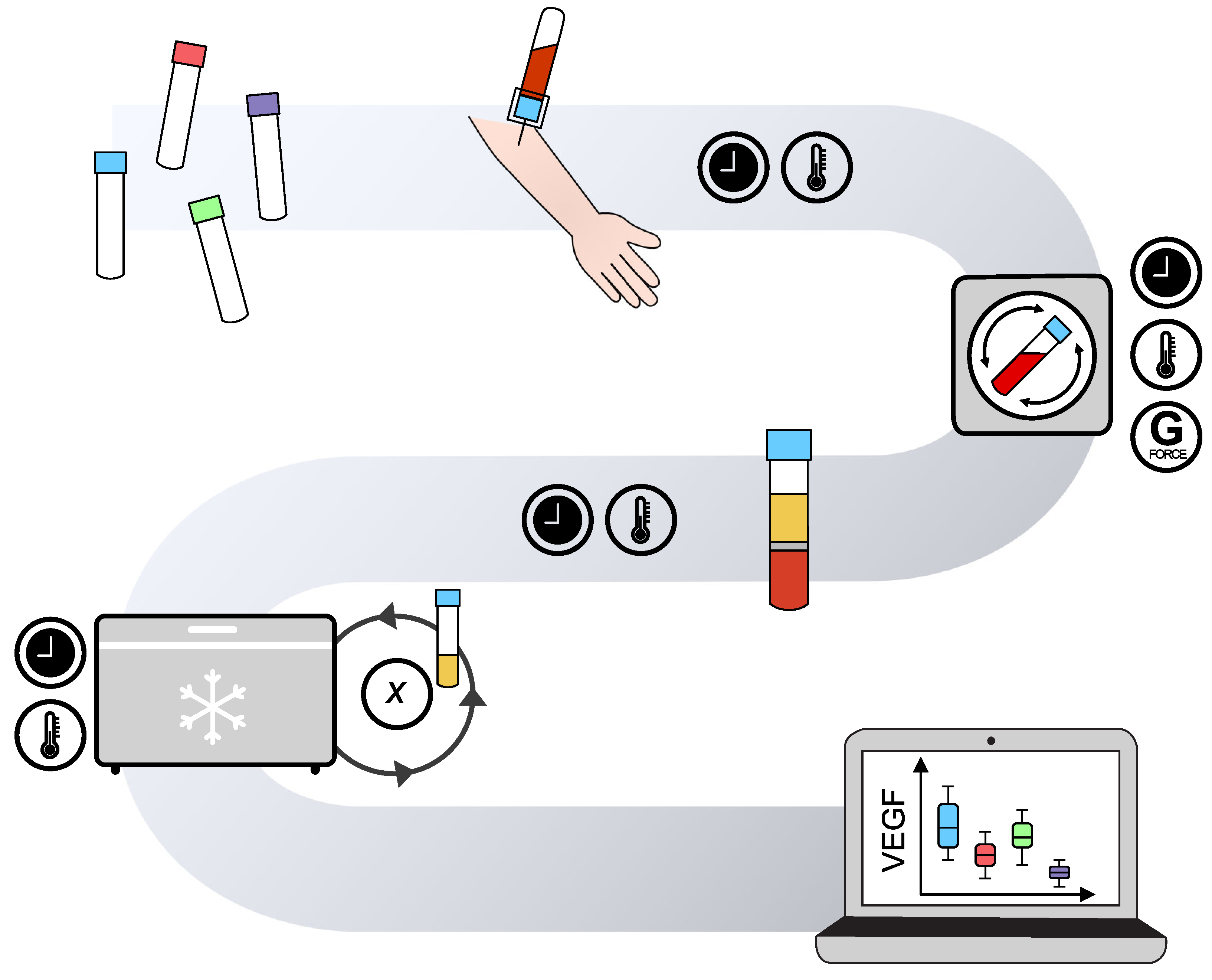

Supplement: S1 Graphical abstract — (TIF) [file pone.0270232.s004.tif]
