# Supplementary material for: A systematic review and meta-analysis of preanalytical factors and methodological differences influencing the measurement of circulating vascular endothelial growth factor
Source: PLoS One. 2022 Jul 6;17(7):e0270232. doi: 10.1371/journal.pone.0270232 (PMC9258884; doi:10.1371/journal.pone.0270232)
Supplement: S1 Appendix — (DOCX) [file pone.0270232.s005.docx]

**Supplementary Appendix to:**

***A Systematic Review and Meta-Analysis of Preanalytical Factors and Methodological Differences Influencing the Measurement of Circulating Vascular Endothelial Growth Factor***

Ulrika Sjöbom, Anders K. Nilsson, Hanna Gyllensten,

Ann Hellström, Chatarina Löfqvist

Contents

[**Risk of bias- Pre-analytical comparison** 3](#_Toc99445238)

[**Methodological comparison** 3](#_Toc99445239)

[**References** 5](#_Toc99445240)

[**S1 Table** 10](#_Toc99445241)

[**S2 Table** 11](#_Toc99445242)

[**S3 Table** 14](#_Toc99445243)

[**S4 Table** 16](#_Toc99445244)

[**S1 Figure** 19](#_Toc99445245)

[**S2 Figure** 20](#_Toc99445246)

# **Risk of bias- Pre-analytical comparison**

Index-test: Twenty-four publications did not report enough information to estimate the risk of bias regarding “the performance of the comparison” [1-24]. For “the conduct and interpretation”, three publications lacked information for both this part and the performance [6, 13, 18], and one study only lacked information for “the conduct and interpretation” [25]. Two publications were estimated as exhibiting a high risk of bias with respect to “the conduct or interpretation of the comparison” [19, 21] . One of these studies was interpreted as having high risk for bias as the interpretation was based on a multiple regression model with several independent variables and few individuals with many replicates. In addition, no formal validation on the multiple regression model was reported [21]. The other study with a high risk of bias for the conduct used a small sample size, and the comparison was not well described [19].

Flow- and timing. Regarding dropouts, 11 publications reported enough information to be assessed for risk of bias; all were considered low risk [3, 5, 12, 15, 20-22, 24, 26-28]. Three studies also gave enough information regarding the flow and the timing; these were assessed as low risk of bias for the flow and timing domain [26-28]. The rest were assessed as unclear for this domain [1-25, 29-39].

# **Methodological comparison**

Only seven included publications compared two or more different analytical methods for the measurement of VEGF levels in blood samples in this systematic review. Four of these studies compared an alternative method with the ELISA assay produced by R&D (Bio-Techne, Minneapolis). Two studies compared home-brew assays with the commercial R&D assay [37, 40]. One study compared a Luminex-based kit obtained from R&D with the ELISA assay [21], and one study compared an AlphaLISA immunoassay obtained from Perkin Elmer [35]. Dupuy et al. [41] compared the Randox Evidence PBAT assay with a Luminex-based method obtained from Merck-Millipore. Two other studies compared a home-brew chemiluminescent assay with a commercial ELISA one used a kit from Miblo Co. [42], and one a kit from Abcam [43]. The reported results are summarized in the S1 Table.

The risk of bias assesment with the Quadas-2 tool for diagnostic accuracy is illustrated in Figure S1. Overall, the risk of bias for patient selection was considered low since all comparisons were performed with the same samples representing the same individuals, thus making the selection of individuals less critical. Regarding the second domain, namely, the “index-test”, the risk of bias was estimated based on “concerns regarding the performance of the analytical and statistical comparison” and “the conduct or interpretation of the comparison”. Five publications did not report enough information to estimate the risk of bias regarding the “performance of the comparison”. For one of those, only minor information was missing regarding “performance of the comparison”, while the “conduct and interpretation” was estimated as low, why the risk of bias for the index test overall was considered low. Three publications lacked information for both the “performance” and “conduct and interpretation”, and one study lacked important information for the “conduct and interpretation”. In total, the risk of bias for the index test was considered low for two publications and unclear for five.

Regarding the reference standard, all included methods and home-brew assays used commercially available VEGF standards, and we assessed the risk of bias as low, as described for the preanalytical results in the manuscript. For the last domain, flow and timing, it was impossible to evaluate the risk of bias since important information was missing in all seven publications.

Comparison between publications showed a good correlation overall but with shifts in VEGF levels. Since the comparisons did not include the same analytical methods, we can not draw any conclusions regarding differences. Nevertheless, disagreements may be likely between methods since heterogeneity within sample systems was reduced when the meta-analysis was performed for the R&D assay only (Figure 4a) compared with the initial meta-analysis with VEGF levels derived from all included healthy individuals (S3 Appendix). Differences between methods have also been described by Jelkmann [44] mainly based on the methodology used for the measurements. The compared method in this systematic review all used immunoassays with antibodies specific for VEGF.

# **References**

1. Aguilar-Mahecha A, Kuzyk MA, Domanski D, Borchers CH, Basik M. The effect of pre-analytical variability on the measurement of MRM-MS-based mid- to high-abundance plasma protein biomarkers and a panel of cytokines. PLoS One. 2012;7(6):e38290. Epub 2012/06/16. doi: 10.1371/journal.pone.0038290. PubMed PMID: 22701622; PubMed Central PMCID: PMCPMC3368926.

2. Azimi-Nezhad M, Lambert D, Ottone C, Perrin C, Chapel C, Gaillard G, et al. Influence of pre-analytical variables on VEGF gene expression and circulating protein concentrations. Biopreserv Biobank. 2012;10(5):454-61. Epub 2012/10/01. doi: 10.1089/bio.2012.0016. PubMed PMID: 24845047.

3. Banks RE, Forbes MA, Kinsey SE, Stanley A, Ingham E, Walters C, et al. Release of the angiogenic cytokine vascular endothelial growth factor (VEGF) from platelets: significance for VEGF measurements and cancer biology. Br J Cancer. 1998;77(6):956-64. Epub 1998/04/07. doi: 10.1038/bjc.1998.158. PubMed PMID: 9528841; PubMed Central PMCID: PMCPMC2150108.

4. Biancotto A, Feng X, Langweiler M, Young NS, McCoy JP. Effect of anticoagulants on multiplexed measurement of cytokine/chemokines in healthy subjects. Cytokine. 2012;60(2):438-46. Epub 2012/06/19. doi: 10.1016/j.cyto.2012.05.019. PubMed PMID: 22705152; PubMed Central PMCID: PMCPMC3449030.

5. Dittadi R, Meo S, Fabris F, Gasparini G, Contri D, Medici M, et al. Validation of blood collection procedures for the determination of circulating vascular endothelial growth factor (VEGF) in different blood compartments. Int J Biol Markers. 2001;16(2):87-96. Epub 2001/07/27. PubMed PMID: 11471901.

6. George ML, Eccles SA, Tutton MG, Abulafi AM, Swift RI. Correlation of plasma and serum vascular endothelial growth factor levels with platelet count in colorectal cancer: clinical evidence of platelet scavenging? Clin Cancer Res. 2000;6(8):3147-52. Epub 2000/08/24. PubMed PMID: 10955796.

7. Hormbrey E, Gillespie P, Turner K, Han C, Roberts A, McGrouther D, et al. A critical review of vascular endothelial growth factor (VEGF) analysis in peripheral blood: is the current literature meaningful? Clin Exp Metastasis. 2002;19(8):651-63. Epub 2003/01/30. doi: 10.1023/a:1021379811308. PubMed PMID: 12553370.

8. Krishnan VV, Ravindran R, Wun T, Luciw PA, Khan IH, Janatpour K. Multiplexed measurements of immunomodulator levels in peripheral blood of healthy subjects: Effects of analytical variables based on anticoagulants, age, and gender. Cytometry B Clin Cytom. 2014;86(6):426-35. Epub 2014/02/28. doi: 10.1002/cyto.b.21147. PubMed PMID: 24574151.

9. Kusumanto YH, Dam WA, Hospers GA, Meijer C, Mulder NH. Platelets and granulocytes, in particular the neutrophils, form important compartments for circulating vascular endothelial growth factor. Angiogenesis. 2003;6(4):283-7. Epub 2004/05/29. doi: 10.1023/B:AGEN.0000029415.62384.ba. PubMed PMID: 15166496.

10. Lee JK, Hong YJ, Han CJ, Hwang DY, Hong SI. Clinical usefulness of serum and plasma vascular endothelial growth factor in cancer patients: which is the optimal specimen? Int J Oncol. 2000;17(1):149-52. Epub 2000/06/15. PubMed PMID: 10853032.

11. Lee JE, Kim SY, Shin SY. Effect of Repeated Freezing and Thawing on Biomarker Stability in Plasma and Serum Samples. Osong public health and research perspectives. 2015;6(6):357-62. Epub 2016/02/03. doi: 10.1016/j.phrp.2015.11.005. PubMed PMID: 26835245; PubMed Central PMCID: PMCPMC4700770.

12. Maloney JP, Silliman CC, Ambruso DR, Wang J, Tuder RM, Voelkel NF. In vitro release of vascular endothelial growth factor during platelet aggregation. Am J Physiol. 1998;275(3):H1054-61. Epub 1998/09/02. doi: 10.1152/ajpheart.1998.275.3.H1054. PubMed PMID: 9724313.

13. McIlhenny C, George WD, Doughty JC. A comparison of serum and plasma levels of vascular endothelial growth factor during the menstrual cycle in healthy female volunteers. Br J Cancer. 2002;86(11):1786-9. Epub 2002/06/28. doi: 10.1038/sj.bjc.6600322. PubMed PMID: 12087467; PubMed Central PMCID: PMCPMC2375407.

14. Ranieri G, Coviello M, Chiriatti A, Stea B, Montemurro S, Quaranta M, et al. Vascular endothelial growth factor assessment in different blood fractions of gastrointestinal cancer patients and healthy controls. Oncol Rep. 2004;11(2):435-9. Epub 2004/01/14. PubMed PMID: 14719080.

15. Salgado R, Benoy I, Bogers J, Weytjens R, Vermeulen P, Dirix L, et al. Platelets and vascular endothelial growth factor (VEGF): a morphological and functional study. Angiogenesis. 2001;4(1):37-43. Epub 2002/02/05. doi: 10.1023/a:1016611230747. PubMed PMID: 11824377.

16. Salven P, Orpana A, Joensuu H. Leukocytes and platelets of patients with cancer contain high levels of vascular endothelial growth factor. Clin Cancer Res. 1999;5(3):487-91. Epub 1999/04/01. PubMed PMID: 10100697.

17. Sanak F, Baenninger P, Kaufmann C, Iselin K, Bachmann L, Buhl D, et al. The Lucerne Protocol for the Production of Autologous Serum Eyedrops. Klin Monbl Augenheilkd. 2021;238(4):346-8. doi: 10.1055/a-1354-6565.

18. Schlingemann RO, Van Noorden CJ, Diekman MJ, Tiller A, Meijers JC, Koolwijk P, et al. VEGF levels in plasma in relation to platelet activation, glycemic control, and microvascular complications in type 1 diabetes. Diabetes Care. 2013;36(6):1629-34. Epub 2013/01/17. doi: 10.2337/dc12-1951. PubMed PMID: 23321217; PubMed Central PMCID: PMCPMC3661842.

19. Starlinger P, Alidzanovic L, Schauer D, Brugger P, Sommerfeldt S, Kuehrer I, et al. Platelet-stored angiogenesis factors: clinical monitoring is prone to artifacts. Dis Markers. 2011;31(2):55-65. Epub 2011/09/08. doi: 10.3233/dma-2011-0798. PubMed PMID: 21896999; PubMed Central PMCID: PMCPMC3826483.

20. Verheul HM, Hoekman K, Luykx-de Bakker S, Eekman CA, Folman CC, Broxterman HJ, et al. Platelet: transporter of vascular endothelial growth factor. Clin Cancer Res. 1997;3(12 Pt 1):2187-90. Epub 1998/11/17. PubMed PMID: 9815613.

21. Walz JM, Boehringer D, Deissler HL, Faerber L, Goepfert JC, Heiduschka P, et al. Pre-Analytical Parameters Affecting Vascular Endothelial Growth Factor Measurement in Plasma: Identifying Confounders. PLoS One. 2016;11(1):e0145375. Epub 2016/01/06. doi: 10.1371/journal.pone.0145375. PubMed PMID: 26730574; PubMed Central PMCID: PMCPMC4711588.

22. b) Werther K, Christensen IJ, Nielsen HJ. Prognostic impact of matched preoperative plasma and serum VEGF in patients with primary colorectal carcinoma. Br J Cancer. 2002;86(3):417-23. Epub 2002/03/05. doi: 10.1038/sj.bjc.6600075. PubMed PMID: 11875709; PubMed Central PMCID: PMCPMC2375229.

23. Wynendaele W, Derua R, Hoylaerts MF, Pawinski A, Waelkens E, de Bruijn EA, et al. Vascular endothelial growth factor measured in platelet poor plasma allows optimal separation between cancer patients and volunteers: a key to study an angiogenic marker in vivo? Ann Oncol. 1999;10(8):965-71. Epub 1999/10/06. doi: 10.1023/a:1008377921886. PubMed PMID: 10509160.

24. Zamudio S, Kovalenko O, Echalar L, Torricos T, Al-Khan A, Alvarez M, et al. Evidence for extraplacental sources of circulating angiogenic growth effectors in human pregnancy. Placenta. 2013;34(12):1170-6. Epub 2013/10/29. doi: 10.1016/j.placenta.2013.09.016. PubMed PMID: 24161217; PubMed Central PMCID: PMCPMC6472272.

25. Hermann N, Dreßen K, Schildberg FA, Jakobs C, Holdenrieder S. Methodical and pre-analytical characteristics of a multiplex cancer biomarker immunoassay. World journal of methodology. 2014;4(4):219-31. Epub 2014/12/30. doi: 10.5662/wjm.v4.i4.219. PubMed PMID: 25541602; PubMed Central PMCID: PMCPMC4274581.

26. Bünger S, Haug U, Kelly M, Posorski N, Klempt-Giessing K, Cartwright A, et al. A novel multiplex-protein array for serum diagnostics of colon cancer: a case–control study. BMC Cancer. 2012;12(1):393. doi: 10.1186/1471-2407-12-393.

27. Brøndum L, Sørensen BS, Eriksen JG, Mortensen LS, Lønbro S, Overgaard J, et al. An evaluation of multiplex bead-based analysis of cytokines and soluble proteins in archived lithium heparin plasma, EDTA plasma and serum samples. Scand J Clin Lab Invest. 2016;76(8):601-11. Epub 2016/09/27. doi: 10.1080/00365513.2016.1230882. PubMed PMID: 27666533.

28. Brookes K, Cummings J, Backen A, Greystoke A, Ward T, Jayson GC, et al. Issues on fit-for-purpose validation of a panel of ELISAs for application as biomarkers in clinical trials of anti-Angiogenic drugs. Br J Cancer. 2010;102(10):1524-32. Epub 2010/04/22. doi: 10.1038/sj.bjc.6605661. PubMed PMID: 20407440; PubMed Central PMCID: PMCPMC2869162.

29. Adams J, Carder PJ, Downey S, Forbes MA, MacLennan K, Allgar V, et al. Vascular endothelial growth factor (VEGF) in breast cancer: comparison of plasma, serum, and tissue VEGF and microvessel density and effects of tamoxifen. Cancer Res. 2000;60(11):2898-905. Epub 2000/06/13. PubMed PMID: 10850435.

30. Guo GH, Dong J, Yuan XH, Dong ZN, Tian YP. Clinical evaluation of the levels of 12 cytokines in serum/plasma under various storage conditions using evidence biochip arrays. Mol Med Rep. 2013;7(3):775-80. Epub 2013/01/08. doi: 10.3892/mmr.2013.1263. PubMed PMID: 23291902.

31. Hetland ML, Christensen IJ, Lottenburger T, Johansen JS, Svendsen MN, Hørslev-Petersen K, et al. Circulating VEGF as a biological marker in patients with rheumatoid arthritis? Preanalytical and biological variability in healthy persons and in patients. Dis Markers. 2008;24(1):1-10. doi: 10.1155/2008/707864. PubMed Central PMCID: PMC18057530.

32. Kisand K, Kerna I, Kumm J, Jonsson H, Tamm A. Impact of cryopreservation on serum concentration of matrix metalloproteinases (MMP)-7, TIMP-1, vascular growth factors (VEGF) and VEGF-R2 in Biobank samples. Clin Chem Lab Med. 2011;49(2):229-35. Epub 2010/12/02. doi: 10.1515/cclm.2011.049. PubMed PMID: 21118050.

33. Larsson A, Sköldenberg E, Ericson H. Serum and plasma levels of FGF-2 and VEGF in healthy blood donors. Angiogenesis. 2002;5(1-2):107-10. Epub 2003/01/29. doi: 10.1023/a:1021588227705. PubMed PMID: 12549867.

34. Licht P, Neuwinger J, Fischer O, Siebzehnrübl E, Wildt L. Peripheral levels of vascular endothelial growth factor (VEGF) are higher in gonadotropin stimulated as compared to natural ovarian cycles. Exp Clin Endocrinol Diabetes. 2001;109(6):345-9. Epub 2001/09/26. doi: 10.1055/s-2001-17402. PubMed PMID: 11571674.

35. Lopez Yomayuza CC, Preissner KT, Lorenz B, Stieger K. Optimizing Measurement of Vascular Endothelial Growth Factor in Small Blood Samples of Premature Infants. Sci Rep. 2019;9(1):6744. Epub 2019/05/03. doi: 10.1038/s41598-019-43108-7. PubMed PMID: 31043645; PubMed Central PMCID: PMCPMC6494810.

36. Svendsen MN, Brünner N, Christensen IJ, Ytting H, Bentsen C, Lomholt AF, et al. Biological variations in plasma VEGF and VEGFR-1 may compromise their biomarker value in colorectal cancer. Scand J Clin Lab Invest. 2010;70(7):503-11. Epub 2010/09/30. doi: 10.3109/00365513.2010.521254. PubMed PMID: 20873967.

37. Webb NJ, Bottomley MJ, Watson CJ, Brenchley PE. Vascular endothelial growth factor (VEGF) is released from platelets during blood clotting: implications for measurement of circulating VEGF levels in clinical disease. Clin Sci (Lond). 1998;94(4):395-404. Epub 1998/06/26. doi: 10.1042/cs0940395. PubMed PMID: 9640345.

38. a) Werther K, Christensen IJ, Nielsen HJ. Determination of vascular endothelial growth factor (VEGF) in circulating blood: significance of VEGF in various leucocytes and platelets. Scand J Clin Lab Invest. 2002;62(5):343-50. Epub 2002/10/22. doi: 10.1080/00365510260296492. PubMed PMID: 12387579.

39. Zhao X, Qureshi F, Eastman PS, Manning WC, Alexander C, Robinson WH, et al. Pre-analytical effects of blood sampling and handling in quantitative immunoassays for rheumatoid arthritis. J Immunol Methods. 2012;378(1-2):72-80. Epub 2012/03/01. doi: 10.1016/j.jim.2012.02.007. PubMed PMID: 22366959; PubMed Central PMCID: PMCPMC3404505.

40. Yang HW, Tsai RY, Chen JP, Ju SP, Liao JF, Wei KC, et al. Fabrication of a Nanogold-Dot Array for Rapid and Sensitive Detection of Vascular Endothelial Growth Factor in Human Serum. ACS applied materials & interfaces. 2016;8(45):30845-52. Epub 2016/11/02. doi: 10.1021/acsami.6b13329. PubMed PMID: 27768268.

41. Dupuy AM, Kuster N, Lizard G, Ragot K, Lehmann S, Gallix B, et al. Performance evaluation of human cytokines profiles obtained by various multiplexed-based technologies underlines a need for standardization. Clin Chem Lab Med. 2013;51(7):1385-93. Epub 2013/01/15. doi: 10.1515/cclm-2012-0648. PubMed PMID: 23314551.

42. Man J, Dong J, Wang Y, He L, Yu S, Yu F, et al. Simultaneous Detection of VEGF and CEA by Time-Resolved Chemiluminescence Enzyme-Linked Aptamer Assay. International journal of nanomedicine. 2020;15:9975-85. Epub 2020/12/29. doi: 10.2147/ijn.S286317. PubMed PMID: 33363367; PubMed Central PMCID: PMCPMC7754089.

43. Ghavamipour F, Rahmani H, Shanehsaz M, Khajeh K, Mirshahi M, Sajedi RH. Enhanced sensitivity of VEGF detection using catalase-mediated chemiluminescence immunoassay based on CdTe QD/H(2)O(2) system. J Nanobiotechnology. 2020;18(1):93. Epub 2020/06/20. doi: 10.1186/s12951-020-00648-9. PubMed PMID: 32552818; PubMed Central PMCID: PMCPMC7302009.

44. Jelkmann W. Pitfalls in the measurement of circulating vascular endothelial growth factor. Clin Chem. 2001;47(4):617-23. Epub 2001/03/29. PubMed PMID: 11274009.

45. SBU. Swedish Agency for Health Technology Assessment and Assessment of Social Services; [updated 2020-10-06; cited 2021 2021-06-28]. Available from: <https://www.sbu.se/globalassets/ebm/bedomning_studier_diagnostisk_tillforlitlighet_quadas2.pdf>.

46. Wade R, Corbett M, Eastwood A. Quality assessment of comparative diagnostic accuracy studies: our experience using a modified version of the QUADAS-2 tool. 2013;4(3):280-6. doi: 10.1002/jrsm.1080.

**S1 Table** Search strategy.

| Data-base | Strategy |
| --- | --- |
| Scopus | TITLE-ABS-KEY ( "Vascular Endothelial Growth Factor A" OR vegf OR vegf-a OR "Vascular Endothelial Growth Factor" OR "Vascular Permeability Factor" ) AND TITLE-ABS-KEY (preanalytical OR preanalytical OR pre-analyze OR preanalyze OR handling OR comparison* OR measurement* OR validation OR standardization OR "blood specimen" OR "specimen handling" OR "blood collection" OR "blood samples" OR "blood sample" OR "platelet activation" OR "platelet stimulation" OR "platelet inhibitor" OR "mechanical stimulation" OR dilution ) AND TITLE-ABS-KEY ( anticoagulants OR anticoagulation OR anticoagulant OR cryopreservation OR freezing OR temperature OR "time factors" OR centrifugation OR centrifuge OR thawing OR storage ) |
| Pubmed | (Vascular Endothelial Growth Factor A[mesh] OR vascular Endothelial Growth Factor A[tiab] OR VEGF[tiab] OR VEGF-A[tiab] OR Vascular Endothelial Growth Factor[tiab] OR Vascular Permeability Factor[tiab]) AND (preanalytical[tiab] OR pre-analytical[tiab] OR pre-analyze[tiab] OR preanalyze[tiab] OR handling[tiab] OR comparison[tiab] OR comparisons[tiab] OR measurement[tiab] OR measurements[tiab] OR validation[tiab] OR standardization[tiab] OR blood specimen collection[mesh] OR blood specimen[tiab] OR specimen handling[mesh] OR specimen handling[tiab] OR blood collection[tiab] OR blood samples[tiab] OR blood sample[tiab] OR platelet activation[tiab] OR platelet stimulation[tiab] OR platelet inhibitor[tiab] OR mechanical stimulation[tiab] OR dilution[tiab]) AND (Anticoagulants[mesh] OR anticoagulation[tiab] OR anticoagulants[tiab] OR anticoagulant[tiab] OR Cryopreservation[mesh] OR cryopreservation[tiab] OR Freezing[mesh] OR freezing[tiab] OR Temperature[mesh] OR temperature[tiab] OR Time Factors[mesh] OR time factors[tiab] OR centrifugation[mesh] OR centrifugation[tiab] OR centrifuge[tiab] OR thawing[tiab] OR storage[tiab]) |

Used terms to perform searches for publications. The searches were conducted without restriction regarding language, dates, or status of journals.

**S2 Table** Data extraction sheet developed based on QUADAS-2 [45, 46].

| **Publication:** |
| --- |
| **Aim:** |
| **Inclusion/exclusion** |
| Does the study investigate how preanalytical factors influence VEGF measurements in blood samples? (inclusion criteria) |
| Are the investigated factors cover biological, environmental, genetic, or physiological variations? (exclusion criteria) |
| Does the study investigate methodological differences for VEGF measurements in blood samples? (inclusion criteria) |
| Does the study report how VEGF concentrations are affected by methodological differences and/or preanalytical handling for at least two groups of comparable samples? (inclusion criteria) |
| Included in review? |
| **General information** |
| Study design |
| Funding or conflict of interest |
| Time for study |
| Country and place for the study |
| Ethics |
| Target condition |
| **1. Patient selection** |
| Describe methods of patient selection |
| Could the selection of patients have introduced bias? * |
| Number of included patients |
| Describe included patients (prior testing, presentation intended use of index test and setting) |
| Number and type of samples used for comparison |
| Is there concern that the included patients do not match the review question?* |
| **2. Index test or method used for comparison** |
| Assay or assays for measurement |
| The specificity of the assay (will be collected from the company that market the assay if not mentioned in the article) |
| How is the experience and the execution of the method used described? (training, established tests, GPC, clinical practice, manufacturer guidance, validation, atypical procedures) |
| How is the performance of the comparison described according to randomization of samples, singlicate/duplicate measurement, etc.? |
| Was the uncertainty in the results reported? Results from the reproducibility for the measurements: intra- and or the inter-assay coefficient of variation |
| Is there concern regarding the performance of the comparison regarding experience, the procedure used, and uncertainty in the results? * |
| Methodological; How was the method comparison performed? Against index test? Independent of each other? |
| Pre-analytical; How was the comparison performed and described? (each sample according to own baseline, groups of samples, etc.) |
| Which comparisons were performed? |
| Statistic methods, levels of significance |
| Results, concentrations, found differences |
| Is there concern that the index test, its conduct, or interpretations differ from the review question?* |
| **3. Reference standard** |
| Was the used method/s calibrated against a reference standard? |
| Could the reference standard, its conduct, or its interpretation have introduced bias? * |
| Methodological; Were the results of both tests verified using the same reference standard? |
| Is there concern that the reference standard's target condition (VEGF measurements) does not match the review question?* |
| **4. Flow and timing** |
| Were all patients included in the comparison? (If not, were they described?) |
| Were all samples included in the comparison? (If not, were there any reason?) |
| Is there concern that dropouts have introduced bias?* |
| Methodological; Did the whole sample undergo both tests (or one test if the study was randomized)? |
| Methodological; Was there a difference in the number of uninterpretable or indeterminate results between tests likely to have biased the study results?  Reasons for such results could be related to test or patient characteristics (possible bias issue) or could be due to chance, for example, equipment failure, which may be common when using developing technology (although this will reduce study power, it is unlikely to be an important bias issue). |
| Methodological; Was there an appropriate interval between the index and the comparator tests? Was the same reagent batch used for each of the included tests? |
| Pre-analytical; Were all samples analyzed using the same method and reagent batch? |
| Pre-analytical; Were the samples measured simultaneously? Were all included individuals analyzed at the same time? |
| Pre-analytical; If measurements were separated in time and batches, was the uncertainty from the inter-assay variance used when interpreting the results? |
| Could the flow and timing in the method comparison have introduced bias? * |
| **The overall risk of bias** |
| 1. Patient selection* |
| 1. Index test* |
| 1. Reference standard* |
| 1. Flow and timing* |

*= risk of bias estimated as low, high, or unclear

**S3 Table** Results from the methodological comparison.

| Study | Method | Number of samples | | Results |
| --- | --- | --- | --- | --- |
| Dupuy et al (2013) | 1. Evidence Investigator® biochip system (Randox, Mauguio, France) 2. Millipore’s Multiplex Cytokine and Chemokine products produce kits for Luminex ® xMAP ® platform. Cat no. MPXHCYTO-60K-19 | 94 | | The number of non-detectable samples was close to zero for both methods. The number of samples above the detection limit was significantly higher for the Luminex assay. For Luminex, 68% was above the detection limit, and for PBAT, 13%, p<0.0001. According to Cohen’s k, clinical concordance was 0.12, indicating a low association between methods based on the number of samples within measuring range with more samples detectable for the Evidence system. Measuring range for Luminex- Millipore: 3.2- 10 000 pg/mL, detection limit 3.2 pg/mL.  Measuring range for Evidence biochip system: 0-3000 pg/mL, detection limit 14.6 pg/mL. No further comparison was reported |
| Ghavamipour et al. (2020) | 1. Conventional Human VEGF ELISA kit, Abcam (Cambridge UK) 2. Home-brew CL-ELISA | Five serum samples | | Linear regression analysis showed excellent correlation between methods, y=0.9988x−6.212, R2=0.997 |
| Lopez et al (2019) | 1. VEGF ELISA DuoSet (R&D systems) 2. AlphaLISA immunoassay (Fa. Perkin Elmer) | | Ten healthy adults with citrate-plasma, serum, recalcified citrate plasma and 6 cord blood samples from preterm infants with citrate plasma and recalcified citrate plasma samples Correlation plots show:  Adults- 6 serum samples and 6 recalcified citrate plasma samples Cord blood- 6 recalcified citrate plasma | The AlphaLISA assay detected lower VEGF-A levels than the R&D assay, but the mean values among the groups were not significantly different between the methods. For cord blood plasma and recalcified serum samples from preterm infants, similar amounts of VEGF-A were quantified with both methods. Pairwise scatter plot analysis in serum and recalcified serum samples from adults and cord blood revealed comparable data. For plasma, some values were below detection level, and comparison in the scatter plot was impossible. AlphaLISA lower detection limit was 2.2 pg/mL, and for R&D 9 pg/mL, intra- and inter-assay precision were below 10% for both methods. |
| Man et al. (2020) | 1. Homebrew chemiluminescent assay, calibrated against VEGF165 from Peprotech, detected by VEGF165 detection probe 2. Human VEGF165 ELISA kit was from Miblo Co. Ltd. (Shanghai, China) | 6 serum samples | | Comparing the methods for VEGF165 concentrations gave a non-significant difference (t = −1.118, P = 0.314) in serum samples. Homebrew method: range 0.5 ng mL−1 and 80 ng mL−1, with the limit of detection 0,1 ng/mL. |
| Walz et al. (2016) | 1. ELISA (R&D) Quantikine assay Cat No. DVE00 2. Luminex- Human VEGF High Sensitivity Kit (R&D) Cat No. LHSCM293 | Six healthy individuals 2 different time-points for sampling and almost 200 samples/aliquots | | The measuring method contributed significantly and independently to explain variation in the multiple regression analysis. Multiplex bead array (Luminex-based) gave lower values compared to ELISA. Median (IQR) VEGF-A concentration for ELISA 36 (19-63) pg/mL and for Luminex 10 (7-18) pg/mL.  Good correlations were found in the scatter plot and Bland-Altman plot. The methods performed equally regarding dilution of extreme values. Further experiments found out that the standard curve produced by the Luminex calibrator resulted in lower concentrations of target VEGF independent of the method. |
| Webb et al. (1998) | 1. R&D VEGF ELISA, mouse monoclonal anti-VEGF, R&D Systems (Abingdon, U.K.). 2. capture by sflt-1 detection with in-house polyclonal anti-VEGF from rabbit against recombinant VEGF156 (Zeneca Pharmaceuticals, Alderley Edge, U.K.) peroxidase-conjugated anti-rabbit (goat) (Jackson ImmunoResearch Luto U.K.) and developed by Amerlite buffer and tablet pack (Berthold UK) | Thirty-four healthy adults and 19 children were undergoing minor surgical procedures under general anesthesia. | | For plasma samples, concentrations compared between the methods were slightly higher for R&D assay (mean [SD] 649 [22.4] pg/ml versus 523 [17.2] pg/ml; unpaired t-test, p = 0.0002). The correlation was r^2^=0.646, p=0.0029. The calibrator from Astra Zeneca produced higher mean values than the R&D recombinant VEGF ratio Astra Zeneca/R&D was 1.24:1. Inter-plate coefficient of variation for sflt-1 detection assay was estimated to be 11.49% and intra-plate variation 4.52%. For R&D, the inter-plate variation was estimated to be 16.1%. The working range was 78 pg/mL-10 ng/mL for the sflt-1 assay,. For R&D ELISA, the sensitivity was reported to 5-10 pg/mL, and the limit of quantification was 31.2 pg/mL for plasma and serum samples. Both assays for VEGF are sensitive to the presence of soluble flt-1 and do not allow detection of VEGF in the form of a fltl-VEGF complex. |
| Yang et al. (2016) | 1. VEGF ELISA (Human VEGF165 Immunoassay, R&D systems) 2. Homebrew- nanogold-dot-array- calibrated against Human VEGF165 PeproTech Inc. (Rocky Hill, NJ, USA) | Three healthy controls and 18 patients with brain tumors. | | The results obtained with the two methods gave no significant differences. |

**S4 Table** Excluded publications.

| Databases and registers (D/R), other methods (O) | Authour (Year) | Title | Journal | Reason for exclusion | Aim | |
| --- | --- | --- | --- | --- | --- | --- |
| O | Belgore F. M., Blann A. D., and LIP G. Y., (2001) | Measurement of free and complexed soluble vascular endothelial growth factor receptor, Flt-1, in fluid samples: development and application of two new immunoassays | Clinical Science | Included no comparison of analytical method, preanalytical procedures or sample system. | | Two novel methods detecting free VEGF and VEGF in complex with sFlt-1 were tested in accordance with standard validation and assessment methodologies employed in commercial settings. They were also applied in a clinical setting. |
| D/R | Choukroun J., and Ghanaati S., (2018) | Reduction of relative centrifugation force within injectable platelet-rich-fibrin (PRF) concentrates advances patients' own inflammatory cells, platelets and growth factors: the first introduction to the low speed centrifugation concept. | [European Journal of Trauma and Emergency Surgery](https://www.springer.com/journal/68/) | Included no comparison of pre-analytical parameters or analytical method on blood samples. | | Analyze systemically the influence of the relative centrifugation force on leukocytes, platelets and growth factor release within fluid platelet-rich fibrin matrices- |
| O | Kakarla V. C., Balaya S., and Murthy R. K., (2010) | ACCURATE ESTIMATION OF VASCULAR ENDOTHELIAL GROWTH FACTOR LEVELS IN MICROSAMPLES WITH A LOW-COST BEAD-BASED ASSAY | Retina, the journal of retinal and vitreous diseases | Included no comparison of pre-analytical parameters or analytical method on blood samples. | | In the study, the accuracy of VEGF measurement with bead-based immunoassay (Luminex) that requires a microsample (50 ␮L) were evaluated and compared with a standard ELISA test using spiked Hank balanced salt solution. |
| O | Karsten E., Breen E., and Herbert B., (2018) | Red blood cells are dynamic reservoirs of cytokines | Scientific Reports | Included no comparison of pre-analytical parameters or analytical method on blood samples. | | The role of RBCs in signalling remains poorly understood. In response, we investigated a panel of 48 cytokines, chemokines, and growth factors that are commonly studied as biomarkers of disease in the analysis of purified RBCs. Importantly, this analysis included identifying the total concentration in RBC lysates and the concentration released by intact RBCs and investigation into how the cytokine profile can be modulated. |
| D/R | Li Y., Wang L., Zhang G-Q., XiaH-Z., and Zou J-P., (2015) | Development and validation of a NANOGold immunoassay for the detection of vascular endothelial growth factor (VEGF) in human serum using inductively coupled plasma mass spectrometry | Chin J Biologicals | Included no comparison of analytical method, preanalytical procedures or sample system. | | Method development and verification of a chemiluminescent immunoassay for VEGF. |
| D/R | Ogge G., Romero R., Kusanovic J. P., Chaiworapongsa T., Dong Z., Mittal O et al., (2010) | Serum and plasma determination of angiogenic and anti-angiogenic factors yield different results: The need for standardization in clinical practice | The Journal of Maternal-Fetal & Neonatal Medicin | Included no measurement of VEGF. | | The purpose of the study was to determine if there are differences in the concentrations of soluble vascular endothelial growth factor receptor, placental growth factor and soluble endoglin between plasma and serum in normal pregnancy and in preeclampsia. |
| O | Ramirez J. S., Bequet-Romero M., Diaz Y. M., Hernández-Bernal F., Santos A de la T., et al. (2018) | Evaluation of methodologies to determine the effect of specific active immunotherapy on VEGF levels in phase I clinical trial patients with advanced solid tumors | Heliyon | Comparison only performed with interference by VEGF immunotherapy. | | The study evaluates the changes on VEGF levels in serum and plasma as well as platelet-derived measurements. Changes in VEGF levels were related with the humoral response seen in cancer patients after an active immunotherapy with a VEGF-based vaccine. |
| D/R | Strandberg G., Sellberg F., Sommar P., Ronaghi M., Lubenow N., Knutsson F., and Berglund D., (2017) | Standardizing the freeze-thaw preparation of growth factors from platelet lysate | Transplantation and cellular engineering | Included no comparison of pre-analytical parameters or analytical method on blood samples. | | Aiming to standardize an optimal method for preparing platelet lysate from plateletphereses. |
| D/R | Thompson D. F., Eborall W., Dinsmore A., Smith C. J., and Duckett C. J., (2010) | Development and validation of a NANOGoldTM immunoassay for the detection of vascular endothelial growth factor (VEGF) in human serum using inductively coupled plasma mass spectrometry | Rapid Communications in Mass Spectrometry | Included no comparison of pre-analytical parameters or analytical method on blood samples. | | The aim of the work was to develop and validate a NANOGold-labelled immunoassay for the detection of VEGF in human serum, which can be routinely used within the clinical setting using detection by ICP-MS. |
| D/R | Walsh D., Sommer G. J., Schaff U. Y., Hahn P. S., Jaffe G. J., and Murthy S. K., (2014) | A centrifugal fluidic immunoassay for ocular diagnostics with an enzymatically hydrolyzed fluorogenic substrate | Lab on a Chip | Included no comparison of pre-analytical parameters or analytical method on blood samples. | | Demonstration of the capability of a novel “Lab-on-a-disk” platform for rapid and sensitive measurement of VEGF for vitreous samples. |

**S1 Figure** Risk of bias estimation according to the QUADAS-2 tool for diagnostic test, based on four domains: 1. Patient selection, 2. Index test, 3. Reference standard and 4. Flow and timing. a) Shows a summary for the seven publications covering a comparison of analytical methods. b) The risk-of-bias assessment for each of the four domains for each of the included publications.


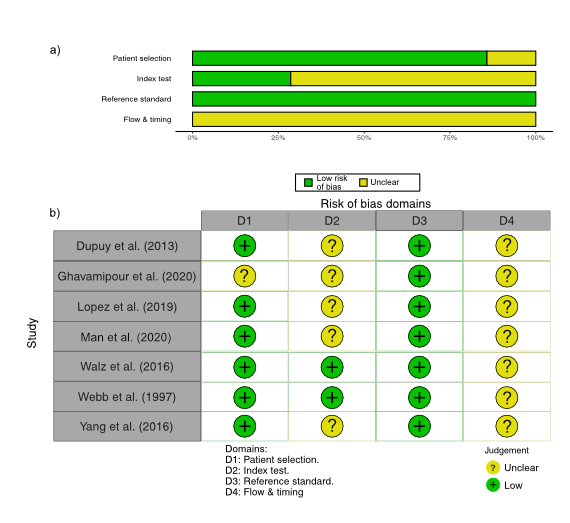


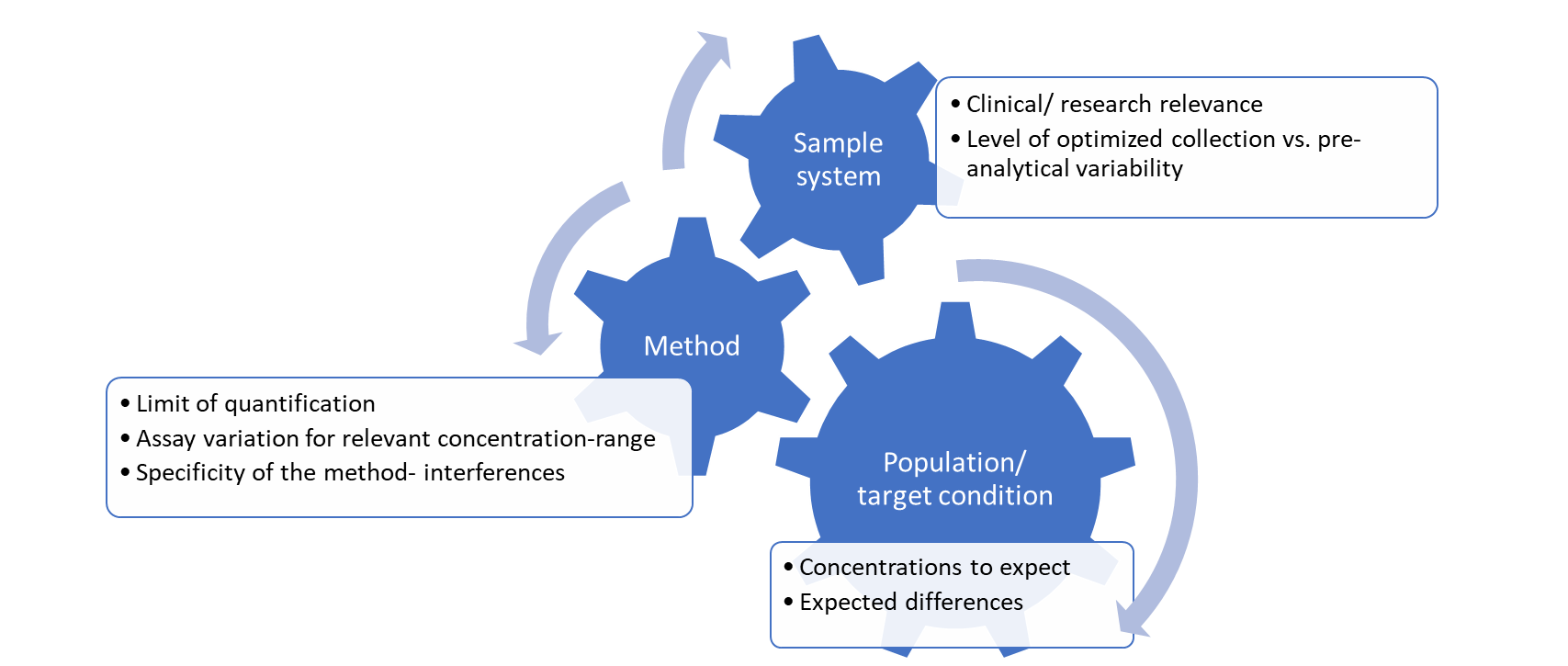


**S2 Figure**

Based on the results in our systematic review, we here list the most critical parameters to consider when deciding on the sample system and method to use for measuring VEGF concentrations.

Start with the **clinical or research relevance.** You have to consider which **sample system** best mirrors disease progression or interesting physiological processes and how earlier studies have been conducted in the same field. Which **level of** **optimized collection** procedures is possible in your study?

Ask questions such as “What is the **limit of quantification** that can be tolerated in our study?”, “What is the **expected concentration range**?”, “What is the **assay variation**?”, “Is the **specificity of the method** important, are there a **risk of interference**, i.e., by hemolytic or lipidemic samples, drugs?”. Such questions are important when deciding which **analytical method** to use.

What is the **expected difference between** the groups that will be compared or **expected** **differences** before and after treatment?

Suppose there is no answer to the questions. In that case, a feasibility study is essential when choosing the analytical method and pre-analytical procedure.
