# Supplementary material for: A systematic review and meta-analysis of preanalytical factors and methodological differences influencing the measurement of circulating vascular endothelial growth factor
Source: PLoS One. 2022 Jul 6;17(7):e0270232. doi: 10.1371/journal.pone.0270232 (PMC9258884; doi:10.1371/journal.pone.0270232)

**Random model meta-analysis of VEGF levels in healthy adults. Means calculated as standardized overall untransformed mean (MRAW) with 95 % confidence.** Sub-groups based on sample system with more than three values: whole blood, serum, activated plasma, EDTA- (including PECT and Edinburgh-plasma), heparin- and citrate-plasma (including, CTAD- and ACD-plasma).

| Source | MRAW (95% CI) | n |
|---|---|---|
| **Whole blood** | | |
| Kusumanto et al. (2004) | 363.14 [ 279.22; 447.06] | 13 |
| Salven et al. (1999) | 303.39 [ 277.85; 328.94] | 56 |
| Werther et al. a) (2002) | 506.00 [ 449.59; 562.41] | 24 |
| Total | 390.06 [ 253.14; 526.97] | **93** |
| Heterogeneity: $\chi^2_2 = 41.45$ ($P < .001$), $I^2 = 95\%$ | | |
| **Serum** | | |
| Adams et al. (2000) | 216.20 [ 180.21; 252.20] | 63 |
| Azimi−Nezhad et al. (2012) | 269.72 [ 205.43; 334.00] | 27 |
| Banks et al. (1998) | 90.00 [ 25.32; 154.68] | 4 |
| Banks et al. (1998) | 284.50 [ 109.95; 459.05] | 8 |
| Biancotto et al. (2012) | 76.90 [ 40.44; 113.36] | 11 |
| Brøndum et al. (2016) | 129.27 [ 77.11; 181.43] | 18 |
| Dittadi et al. (2001) | 289.90 [ 128.13; 451.67] | 10 |
| George et al. (2000) | 190.29 [ 152.15; 228.43] | 74 |
| Guo et al. (2013) | 128.09 [ 78.78; 177.40] | 9 |
| Guo et al. (2013) | 134.22 [ 83.09; 185.36] | 9 |
| Hetland et al. (2008) | 305.52 [ 225.38; 385.66] | 10 |
| Hetland et al. (2008) | 358.79 [ 324.89; 392.70] | 306 |
| Krishnan et al. (2014), age group 18−30 | 6.40 [ 5.41; 7.39] | 31 |
| Krishnan et al. (2014), age group 31−50 | 14.30 [ 8.04; 20.56] | 30 |
| Larsson et al (2001) | 159.70 [ 132.05; 187.35] | 80 |
| Lee et al. (2000) | 232.40 [ 182.92; 281.88] | 42 |
| Lopez Yomayuza et al. (2019) | 222.70 [ 143.92; 301.48] | 10 |
| Lopez Yomayuza et al. (2019) | 278.14 [ 260.87; 295.41] | 10 |
| Lopez Yomayuza et al. (2019) | 129.60 [ 62.20; 197.00] | 10 |
| Maloney et al. (1998) | 230.00 [ 190.95; 269.05] | 10 |
| McIlhenny et al. (2002) | 299.55 [ 229.60; 369.50] | 20 |
| Ranieri et al. (2004) | 383.00 [ 270.30; 495.70] | 16 |
| Salgado et al. (2001) | 97.20 [ 68.70; 125.70] | 24 |
| Salven et al. (1999) | 117.30 [ 66.45; 168.15] | 23 |
| Starlinger et al. (2011) | 631.10 [ 422.66; 839.54] | 3 |
| Verheul et al. (1997) | 285.18 [ 82.87; 487.49] | 30 |
| Webb et al. (1997) | 249.40 [ 158.46; 340.34] | 34 |
| Webb et al. (1997) | 281.29 [ 168.56; 394.03] | 6 |
| Webb et al. (1997) | 239.16 [ 146.16; 332.15] | 10 |
| Werther et al. a) (2002) | 391.00 [ 320.99; 461.01] | 24 |
| Werther et al. b) (2002) | 271.66 [ 213.92; 329.40] | 50 |
| Wynendael et al. (1999) | 195.71 [ 144.15; 247.27] | 22 |
| Total | 211.05 [ 175.66; 246.43] | **1034** |
| Heterogeneity: $\chi^2_{31} = 2684.72$ ($P < .001$), $I^2 = 99\%$ | | |
| **Activated citrate plasma** | | |
| Banks et al. (1998) | 105.38 [ 28.11; 182.64] | 8 |
| Banks et al. (1998) | 148.62 [ 43.05; 254.20] | 8 |
| Lopez Yomayuza et al. (2019) | 161.10 [ 105.50; 216.70] | 10 |
| Lopez Yomayuza et al. (2019) | 194.43 [ 170.27; 218.60] | 10 |
| Lopez Yomayuza et al. (2019) | 102.54 [ 31.90; 173.18] | 10 |
| Ranieri et al. (2004) | 197.00 [ 105.86; 288.14] | 16 |
| Ranieri et al. (2004) | 77.00 [ 35.84; 118.16] | 16 |
| Webb et al. (1997) | 127.96 [ 68.61; 187.31] | 6 |
| Total | 138.85 [ 98.11; 179.59] | **84** |
| Heterogeneity: $\chi^2_7 = 29.4$ ($P < .001$), $I^2 = 76\%$ | | |
| **EDTA-plasma** | | |
| Azimi−Nezhad et al. (2012) | 135.72 [ 109.95; 161.48] | 27 |
| Banks et al. (1998) | 60.00 [ 15.90; 104.10] | 4 |
| Biancotto et al. (2012) | 4.70 [ 1.76; 7.64] | 11 |
| Brøndum et al. (2016) | 31.46 [ 17.88; 45.04] | 18 |
| Brøndum et al. (2016) | 89.01 [ 46.16; 131.86] | 15 |
| Dittadi et al. (2001) | 6.10 [ 3.93; 8.27] | 10 |
| Hetland et al. (2008) | 20.68 [ 8.39; 32.96] | 10 |
| Hetland et al. (2008) | 51.97 [ 45.28; 58.66] | 306 |
| Krishnan et al. (2014), age group 18−30 | 33.70 [ 10.55; 56.85] | 30 |
| Krishnan et al. (2014), age group 31−50 | 15.70 [ 12.36; 19.04] | 31 |
| Krishnan et al. (2014), age group 51−64 | 25.60 [ 19.37; 31.83] | 30 |
| Krishnan et al. (2014), age group 65−85 | 14.50 [ 8.67; 20.33] | 16 |
| Larsson et al (2001) | 32.20 [ 27.42; 36.98] | 80 |
| Lee et al. (2000) | 48.20 [ 37.71; 58.69] | 42 |
| McIlhenny et al. (2002) | 14.57 [ 8.67; 20.48] | 20 |
| Schlingemann et al. (2013) | 6.10 [ 2.08; 10.12] | 21 |
| Starlinger et al. (2011) | 29.60 [ 10.59; 48.61] | 3 |
| Verheul et al. (1997) | 42.40 [ 4.77; 80.03] | 30 |
| Walz et al. (2016) | 16.80 [ 9.75; 23.85] | 6 |
| Walz et al. (2016) | 70.54 [ 22.61; 118.47] | 6 |
| Walz et al. (2016) | 60.60 [ 33.44; 87.76] | 8 |
| Walz et al. (2016) | 7.67 [ 3.96; 11.38] | 2 |
| Walz et al. (2016) | 10.08 [ 0.87; 19.29] | 2 |
| Walz et al. (2016) | 21.90 [ 15.66; 28.14] | 8 |
| Webb et al. (1997) | 76.10 [ 55.13; 97.07] | 34 |
| Webb et al. (1997) | 108.30 [ 85.76; 130.84] | 20 |
| Werther et al. a) (2002) | 63.00 [ 40.20; 85.80] | 24 |
| Werther et al. b) (2002) | 57.10 [ 34.36; 79.84] | 50 |
| Wynendael et al. (1999) | 15.91 [ 9.46; 22.36] | 22 |
| Total | 33.30 [ 27.20; 39.40] | **886** |
| Heterogeneity: $\chi^2_{28} = 623.66$ ($P < .001$), $I^2 = 96\%$ | | |
| **Heparin-plasma** | | |
| Banks et al. (1998) | 28.00 [ 5.46; 50.54] | 4 |
| Biancotto et al. (2012) | 23.00 [ 14.38; 31.62] | 11 |
| Brøndum et al. (2016) | 51.78 [ 32.10; 71.45] | 15 |
| Guo et al. (2013) | 9.64 [ 6.35; 12.93] | 9 |
| Hetland et al. (2008) | 33.66 [ 22.39; 44.94] | 10 |
| Krishnan et al. (2014), age group 18−30 | 17.40 [ 12.18; 22.62] | 30 |
| Maloney et al. (1998) | 38.20 [ 33.43; 42.97] | 10 |
| Total | 27.60 [ 16.28; 38.93] | **89** |
| Heterogeneity: $\chi^2_6 = 111.48$ ($P < .001$), $I^2 = 95\%$ | | |
| **Citrate-plasma** | | |
| Adams et al. (2000) | 30.77 [ 26.44; 35.09] | 63 |
| Azimi−Nezhad et al. (2012) | 69.57 [ 55.41; 83.73] | 27 |
| Banks et al. (1998) | 18.00 [ 1.34; 34.66] | 4 |
| Banks et al. (1998) | 22.14 [ 14.73; 29.55] | 8 |
| Biancotto et al. (2012) | 9.70 [ 2.06; 17.34] | 11 |
| Dittadi et al. (2001) | 26.70 [ 8.48; 44.92] | 10 |
| Dittadi et al. (2001) | 10.70 [ 4.94; 16.46] | 10 |
| George et al. (2000) | 6.72 [ 5.84; 7.59] | 113 |
| Hetland et al. (2008) | 24.69 [ 17.24; 32.14] | 10 |
| Hormbrey et al. (2002) | 37.40 [ 27.42; 47.38] | 8 |
| Hormbrey et al. (2002) | 32.14 [ 23.76; 40.52] | 8 |
| Krishnan et al. (2014), age group 18−30 | 20.30 [ 7.88; 32.72] | 30 |
| Krishnan et al. (2014), age group 31−50 | 9.70 [ 7.24; 12.16] | 31 |
| Kusumanto et al. (2004) | 9.95 [ 6.23; 13.66] | 13 |
| Lopez Yomayuza et al. (2019) | 38.00 [ 5.58; 70.42] | 10 |
| Lopez Yomayuza et al. (2019) | 55.09 [ 22.98; 87.20] | 10 |
| Lopez Yomayuza et al. (2019) | 9.17 [ 5.22; 13.11] | 10 |
| Ranieri et al. (2004) | 21.00 [ 12.67; 29.33] | 16 |
| Ranieri et al. (2004) | 33.00 [ 3.11; 62.89] | 16 |
| Salgado et al. (2001) | 13.70 [ 10.02; 17.38] | 24 |
| Schlingemann et al. (2013) | 10.40 [ 8.30; 12.50] | 21 |
| Starlinger et al. (2011) | 10.40 [ 2.37; 18.43] | 3 |
| Starlinger et al. (2011) | 3.13 [ 1.66; 4.60] | 13 |
| Starlinger et al. (2011) | 9.28 [ 5.00; 13.56] | 10 |
| Starlinger et al. (2011) | 14.30 [ 2.87; 25.73] | 3 |
| Walz et al. (2016) | 3.80 [ 0.53; 7.07] | 2 |
| Walz et al. (2016) | 11.96 [ 4.59; 19.33] | 2 |
| Walz et al. (2016) | 19.60 [ 15.51; 23.69] | 8 |
| Webb et al. (1997) | 35.53 [ 9.78; 61.28] | 6 |
| Wynendael et al. (1999) | 6.05 [ 2.73; 9.36] | 22 |
| Wynendael et al. (1999) | 19.47 [ 8.25; 30.69] | 10 |
| Total | 16.70 [ 13.82; 19.57] | **532** |
| Heterogeneity: $\chi^2_{30} = 427.04$ ($P < .001$), $I^2 = 93\%$ | | |
| Total | 55.70 [ 51.66; 59.75] | **2718** |
| Heterogeneity: $\chi^2_{109} = 5188.18$ ($P < .001$), $I^2 = 98\%$ | | |
| Test for subgroup differences: $\chi^2_5 = 196.12$ ($P < .001$) | | |

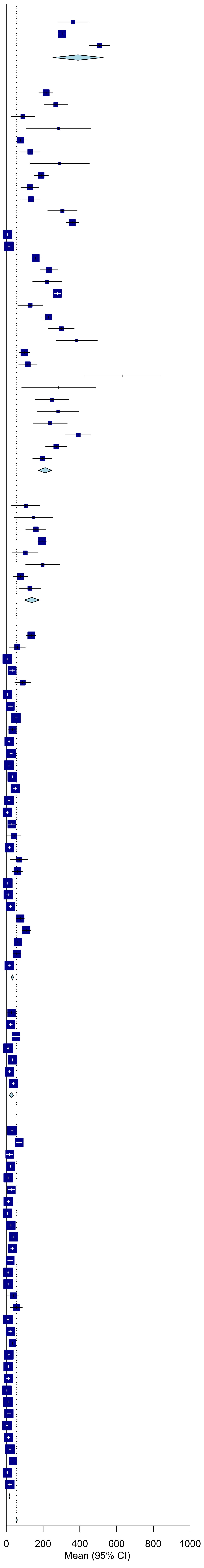

Supplement: S3 Appendix — (PDF) [file pone.0270232.s007.pdf]
